# The Influence of Short Motifs on the Anticancer Activity of HB43 Peptide

**DOI:** 10.3390/pharmaceutics14051089

**Published:** 2022-05-19

**Authors:** Claudia Herrera-León, Francisco Ramos-Martín, Hassan El Btaouri, Viviane Antonietti, Pascal Sonnet, Laurent Martiny, Fabrizia Zevolini, Chiara Falciani, Catherine Sarazin, Nicola D’Amelio

**Affiliations:** 1 Unité de Génie Enzymatique et Cellulaire UMR 7025 CNRS, Université de Picardie Jules Verne, 80039 Amiens, France; claudia.herrera@u-picardie.fr (C.H.-L.); francisco.ramos@u-picardie.fr (F.R.-M.); catherine.sarazin@u-picardie.fr (C.S.); 2 Matrice Extracellulaire et Dynamique Cellulaire UMR 7369 CNRS, Université de Reims Champagne Ardenne (URCA), 51100 Reims, France; hassan.elbtaouri@univ-reims.fr (H.E.B.); laurent.martiny@univ-reims.fr (L.M.); 3 Agents Infectieux, Résistance et Chimiothérapie, AGIR UR 4294, Université de Picardie Jules Verne, UFR de Pharmacie, 80037 Amiens, France; viviane.silva-pires@u-picardie.fr (V.A.); pascal.sonnet@u-picardie.fr (P.S.); 4 Department of Medical Biotechnology, University of Siena, 53100 Siena, Italy; fabrizia.zevolini@student.unisi.it (F.Z.); chiara.falciani@unisi.it (C.F.)

**Keywords:** anticancer, antimicrobial, peptides, sequence alignment, biomembranes, NMR, molecular dynamics

## Abstract

Despite the remarkable similarity in amino acid composition, many anticancer peptides (ACPs) display significant differences in terms of activity. This strongly suggests that particular relative dispositions of amino acids (motifs) play a role in the interaction with their biological target, which is often the cell membrane. To better verify this hypothesis, we intentionally modify HB43, an ACP active against a wide variety of cancers. Sequence alignment of related ACPs by ADAPTABLE web server highlighted the conserved motifs that could be at the origin of the activity. In this study, we show that changing the order of amino acids in such motifs results in a significant loss of activity against colon and breast cancer cell lines. On the contrary, amino acid substitution in key motifs may reinforce or weaken the activity, even when the alteration does not perturb the amphipathicity of the helix formed by HB43 on liposomes mimicking their surface. NMR and MD simulations with different membrane models (micelles, bicelles, and vesicles) indicate that the activity reflects the insertion capability in cancer-mimicking serine-exposing membranes, supported by the insertion of N-terminal phenylalanine in the FAK motif and the anchoring to the carboxylate of phosphatidylserine by means of arginine side chains.

## 1. Introduction

Although much progress has been made, cancer therapies still suffer from the development of resistance, as well as the severe side effects caused by the low specificity of currently available drugs [1]. Recent years have witnessed the rise of new classes of molecules promising to overcome these concerns. Among these, anticancer peptides (ACPs) have shown great potential as novel therapeutic agents due to several interesting properties including broad-spectrum activity, short-term action decreasing the probability of resistance, good solubility allowing efficient tissue penetration, and specificity for cancer cells resulting in reduced side effects [2,3,4,5,6,7]. The emphasis on peptide drug development has resulted in hundreds of new ACPs that have grown this research niche. Until now, 3500 ACPs have been described in the CancerPPD database [8]. It is estimated that the global peptide drug market will be worth around USD 48.04 billion by 2025 [9,10].

It is believed that ACPs act by destabilizing plasma membranes [11,12,13,14,15], explaining their selectivity towards cancer cells. This is due to an intrinsic difference between the membranes of most non-cancerous and cancerous cells, in terms of charge and fluidity. As opposed to the essentially uncharged human cell membranes, cancer cell membranes tend to be more negatively charged in their outer leaflet because of the exposition of sialic acid-rich glycoproteins, phosphatidylserine (PS), or heparan sulfate [2]. The glycosylation profile of cancer cells has been extensively studied and it was shown that defects in selected enzymes lead to a well-defined glycosylation profile and overproduction of negatively charged sialic acid [16]. Another important issue is membrane fluidity which is strongly influenced by cholesterol (Cho) content [17,18]. Cancer cells have been reported to be more fluid, due to a reduced presence of Cho, allowing them to enter blood vessels easier [18,19], but impeding ACPs to enter human cells [17,20]. Indeed, malignant potential and capability of metastasis are highly correlated with membrane deformations and Cho levels [17].

The promising future of this research niche is evident, but there is an urgent need to design a strategy that addresses the limitations associated with ACPs, such as high cost of production and instability in blood plasma [21,22]. The genetic engineering of the microbiome for the expression of ACPs has been used successfully to overcome the problems associated with the production costs and provide continuous in-situ surveillance and protection from tumorigenesis [23,24]. Other technologies include the encapsulation in nanogels that allows delivery of ACPs to the desired cell target while protecting them from the action of degrading enzymes and controlling their release [25,26,27,28].

Computational design is also a powerful tool to create new peptides with favorable therapeutic indexes [9,29]. For example, many peptides have been rationally designed using the scaffold of the existing ACPs [30] or planning chemical modifications such as cyclization, acetylation, or the use of D-amino acids [31,32]. Liu and colleagues (2011) investigated whether the conjugation of magainin II (MG2, GIGKFLHSAKKFGKAFVGEIMNS) to the tumor-homing peptide bombesin (QRLGNQWAVGHLM) could improve its activity towards cancer cells. Indeed, the conjugate showed higher cytotoxicity against cancer cells in vitro and reduced tumor weights in mice with MCF-7 tumor grafts in vivo [33]. Papo and colleagues (2006) showed how the modified peptide D-K6L9 (LK*L*LKKL*L*KKLL*K*LL-NH_2_, italic letters are D-amino acids) prevented the metastatic spread of 22R*v1* prostate carcinoma and MDA-MB231 breast cancer cells. The high specificity to cancer cells was attributed to its binding to the anionic surface-exposed PS followed by depolarization of the cytoplasmic membrane leading to cell death [34].

Some of these constructs have been patented. It is the case of FLAK peptides, a family of short-length peptides (no more than 23 amino acids) that contain only phenylalanine, leucine, alanine, and lysine (hence the name FLAK) [35,36]. In some cases, the N-terminus might be acetylated and the C-terminus amidated. The alternation of charged and hydrophobic residues suggests amphipathicity [34,37,38] and an alpha-helical structure. FLAK peptides show antibacterial (Gram-positive and Gram-negative) [37,38,39,40], antifungal [40], antiviral [41], and anticancer activities, the latter expressed as LD50, between 2 and 91 µg/mL acting on cancer cell lines HeLa [12,39], MCF-7 [37], SW-480 [39], A549, PC-3 [37], CL1, 22R*v1*, MB-231 [34] derived from breast, colon, lung, prostate, and cervical cancer.

Despite a remarkable similarity in amino acid composition, they display significant differences in terms of activity. This strongly suggests that particular relative dispositions of amino acids (motifs) play a role in the recognition process. To better understand this effect, we decided to study the HB43 peptide (also called FLAK50). This peptide, with sequence FAKLLAKLAKKLL, has been shown to have an alpha structure and affinity for phosphatidylserine [42]. Furthermore, it has been reported to be active on skin, lung, cervical, breast, colon, and prostate cancer, according to the CancerPPD database [8]. HB43 also displays antibacterial properties against *Pseudomonas aeruginosa* in cystic fibrosis (CF) [40,43] and *Staphylococcus aureus* in catheter-related infection (CRI) [44].

Studies on the structure-activity relationship are crucial to better understand its biological activity and improve its efficacy. For this, NMR is one of the most suitable techniques, since it allows obtaining structural and dynamic information at the atomic level on both the peptide and the target membrane (which can be mimicked by various phospholipid models). In this work, we show by various biophysical and in silico approaches how mutations altering recurrent short motifs in the HB43 sequence-related family of ACPs affect the anticancer activity. Furthermore, the effect reflects the degree of insertion in model membranes rich in PS, usually found in the external layer of apoptotic cancer cells [45].

## 2. Materials and Methods

### 2.1. Sequence Alignment by ADAPTABLE Web Server

A general family of anticancer peptides was created by the family generator page of ADAPTABLE web server (http://gec.u-picardie.fr/adaptable (accessed on 10 February 2021)) with the following parameters [46]: “anticancer” = “y”; “anticancer activity (µM)” = 10; “Substitution matrix” = “Unitary”; “Minimum % of similarity” = 0; “Generate additional graphical analysis” = “y”. Out of the many families created, Family 1 contains 298 peptides found in the ADAPTABLE database.

The family of peptides sequence-related to HB43 (FAKLLAKLAKKLL) was created by the family generator page of ADAPTABLE webserver using “Create the family of a specific peptide” option with the following parameters: “anticancer” = “y”; “anticancer activity (µM)” = 10; “Substitution matrix” = “Blosum45”; “Minimum % of similarity” = 70. As ADAPTABLE continuously updates with new entries, sequence-related families might change slightly with time [46].

### 2.2. Peptide Synthesis

Fmoc(9-fluorophenylmethoxy)-amino acids, Fmoc-Tyr(*t*Bu)-AC TentaGel^®^ resin (0.22 mmol/g, particle size: 90 μm) and Fmoc-TentaGel^®^-S RAM resin (0.24 mmol/g, particle size: 90 μm) were purchased from Iris Biotech GmbH (Marktredwitz, Germany). The other chemical compounds were purchased from VWR Chemicals, Iris Biotech, or Acros and used without further purification. Peptides were synthesized on a CEM Liberty 1 Microwave Peptide Synthesizer (CEM Corporation, Matthews, NC, USA), using standard automated continuous-flow microwave solid-phase peptide synthesis methods. A five-fold molar excess of the above amino acids was used in a typical coupling reaction. Fmoc-deprotection was accomplished by treatment with 20% (*v*/*v*) piperidine in N-methyl-2-pyrrolidone (NMP) at 75 °C. The coupling reaction was achieved by treatment with 2-(1H-benzotriazol-1-yl)-1,1,3,3-tetramethyluronium hexafluorophosphate (HBTU) and N,N-diisopropylethylamine (DIEA) in NMP using a standard microwave protocol (75 °C). Peptides were cleaved and sidechains deprotected by treatment of the peptide resin with a mixture of 1.85 mL of trifluoroacetic acid (TFA), 50 μL of triisopropylsilane, 50 μL H_2_O and 50 mg of DL-dithiothreitol, in respective percent proportions, 92.5/2.5/2.5/2.5, during 4 h at room temperature. The solid support was removed by filtration, the filtrate concentrated under reduced pressure, and the peptide precipitated from diethyl ether. The precipitate was washed several times with diethyl ether and dried under reduced pressure. Peptides were purified on an RP-HPLC C18 column (Phenomenex^®^ C18, Jupiter 4μ Proteo, 90 Å, 250 × 21.20 mm) using a mixture of aqueous 0.1% (*v*/*v*) TFA (A) and 0.1% (*v*/*v*) TFA in acetonitrile (B) as the mobile phase (flow rate of 3 mL/min) and employing UV detection at 210 and 254 nm. The purity of all peptides was found to be >95% (Figures S11–S14).

### 2.3. Cell Cultures

Human colorectal cancer cell lines SW620 (ATCC, #CCL-227, isolated from the large intestine of a 51-year-old male Dukes C colorectal cancer patient), SW480 (ATCC, #CCL-228, isolated from the large intestine of a male Dukes B colorectal cancer patient), and HT29 (ATCC, #HTB-38, isolated from a white, female colorectal adenocarcinoma patient) were purchased from American Type Culture Collection (ATCC, Rockville, MD, USA). They were cultured in 25 cm^2^ flasks containing Dulbecco’s Modified Eagle’s Medium (DMEM 1× Glutamax; Gibco, Fisher Scientific, Illkirch, France) supplemented with 10% (*v*/*v*) fetal bovine serum (FBS) and 100 µg/mL streptomycin. Culture flasks (Falcon, Milan, Italy) containing cells were incubated at 37 °C in 5% CO_2_. After trypsinization, cells were cultured in 96-well plates for viability assay and in 12-well plates for flow cytometry and zymography.

### 2.4. Cell Viability Assay

SW480, SW620, and HT29 cells were seeded into 96-well plates at a density of 8 × 10^6^ cells/mL for 24 h at 37 °C in 5% CO_2_. The culture medium was then gently removed and 200 µL of each peptide at different concentrations (2.5, 5, 10, 15, 25, and 50 µM) were added to the wells. Control cells were treated with a culture medium without peptides. After 24 h and 48 h of incubation at 37 °C in 5% CO_2_, the culture medium was gently removed and the cells were exposed to AlamarBlue 10% (*v*/*v*) in PBS and incubated for 2 h at 37 °C in 5% CO_2_. Absorbance was then measured at a wavelength of 560–600 nm on a microplate reader (TECAN, Infinite, Männedorf, Switzerland). Cell viability was calculated as a percentage difference in reduction between treated and control cells. Each peptide concentration was analyzed in triplicate, and the experiment was repeated 3 times with each cell line.

### 2.5. Gelatin Zymography

Zymography assay was used to detect matrix metalloproteinase activity. Cells in serum-free medium were seeded into 96-well plates at a density of 2 × 10^5^ cells/mL and incubated for 24 h at 37 °C in 5% CO_2_. The medium was removed and cells were treated with 5 µM and 20 µM of peptides HB43, *mut2*, and *mut3*, respectively. After incubation, the medium was collected and samples were run under non-reducing conditions in 10% polyacrylamide [sodium dodecyl sulfate (SDS) 0.1%] gels containing gelatin (1%). SDS was removed by washing twice at room temperature in 2.5% Triton X-100 solution. The metalloproteinase (MMP) activity was reactivated by incubating the gel in a buffer containing Tris-NaCl 50 mM pH 7.4, CaCl_2_ 5 mM, and Triton X-100 at 0.1% (*w*/*v*), for 24 h at 37 °C. Gels were stained with Coomassie Blue in a 10% acetic acid, 45% methanol solution for 45 min, and then, distained a first time in a 10% acetic acid, 25% methanol solution for 45 min, and a second time in 5% glycerol, 5% methanol for 45 min. MMP activity was detected as clear bands present on the blue background where MMPs had digested the gelatin substrate.

### 2.6. Flow Cytometry Analysis of Cell Cycle

Cells were seeded into 96-well plates at a density of 5 × 10^5^ cells/mL and incubated for 24 h in a medium containing 10% FBS at 37 °C in 5% CO_2_. Then, cells were treated with a medium containing 5 µM of peptides HB43, *mut3,* and 20 µM of *mut2* and incubated for 12 h at 37 °C. Cells were washed with PBS and a hypotonic propidium iodide citrate stain (50 μg/mL in 0.1% trisodium citrate dihydride) containing 0.3 μL/mL of Nonidet P-40 was added. Finally, the adherent cells were harvested by scraping vigorously with a tip of a pipette to dislodge any cytoplasmic remnants from the lysed cells and kept at 4 °C until analysis. The stained cells were analyzed using a BD Accuri™ C6 flow cytometer integrated with a BD CSampler (BD Biosciences, San Jose, CA, USA).

### 2.7. Statistical Analysis

Data were expressed as the mean and standard deviation (S.D.). Cell viability was performed in triplicate. Flow cytometry analysis was performed in duplicate and data were analyzed with a *t*-test. All statistical analyses were performed with GraphPad Prism 9. *p*-values < 0.05 were considered statistically significant.

### 2.8. Sample Preparation

Samples for liquid NMR were prepared by hydration of a 0.8 mM peptide sample in 500 µL of 10 mM phosphate buffer at pH 6.6 containing 10% of D_2_O as a field-locking signal and 2 µL of 100 µM deuterated sodium 3-(trimethylsilyl)propionate-d4 (TSP-d4). For assignment and determination of the secondary structure, a 1M solution of Dodecylphosphocholine-d38 (DPC:d38) was prepared in a 10 mM phosphate buffer at pH 6.6. Then, a 0.8 mM peptide sample was titrated with the concentrated solution of DPC micelles until reaching a final concentration of 50 mM.

Isotropic bicelles for solution NMR were prepared as follows. A mixture of 33.3% DMPC (1,2-dimyristoyl-sn-glycero-3-phosphocholine) and 66% DHPC (1,2-dihexanoyl-sn-glycero-3-phosphocholine) in chloroform was used to obtain a molar ratio of 0.5 (q = ([DMPC]/[DHPC]) thus ensuring a small almost spherical morphology [42]. The solvent was removed under a nitrogen stream and the samples were then lyophilized and resuspended in a 10 mM phosphate buffer at pH 6.6, to reach a final concentration of 1 M for the stock solution. Additionally, 1.6 mM samples of peptides HB43, *mut2*, and *mut3* (90% 10 mM phosphate buffer/10% D_2_O) were titrated with bicelles up to a final lipid concentration of 100 mM.

Small unilamellar vesicles (SUVs) were prepared by sonication of a vesicle suspension containing non-deuterated lipids (50%:50% POPC (1-palmitoyl-2-oleoyl-glycero-3-phosphocholine)/POPS (1-palmitoyl-2-oleoyl-sn-glycero-3-phospho-l-serine)) in an adequate amount to obtain a total lipid concentration of 20 mM. Sonication was performed using a probe-type sonicator at 33 W in an ice bath until a clear solution was obtained (cycling for 30 min in 10-min intervals with 12-s ON and 2-s OFF pulses). Suspensions were afterward centrifuged at 60,000× *g* for 5 min to remove metallic particles and the clear supernatant was used for experiments.

Multilamellar vesicles (MLVs) for ssNMR studies were prepared using phospholipids deuterated at the level of the palmitoyl chain, according to the conventional protocol [47,48,49,50]. Briefly, phospholipids were solubilized in chloroform, and solutions were mixed to obtain the desired proportions in a total lipid amount of 60 mM. In particular, we used 50%:50% POPC/POPC:d31, 50%:50% POPS/POPS:d31 and 50%:50% POPC/POPS:d31. The solvent was then removed under a nitrogen stream. Samples were hydrated with ultrapure water, well-vortexed to promote total hydration, and lyophilized overnight to remove solvent traces. The resulting powder containing lipids was hydrated by 80 µL of ultrapure water (for non-charged lipids) or 10 mM phosphate buffer at pH 6.6 and 100 mM in NaCl (for charged lipids) and homogenized by shaking. Four freeze-thaw cycles were applied: one step of freezing (−80 °C, 15 min) followed by thawing (40 °C, 15 min) and shaking for 1 min every two cycles. Finally, MLV samples were placed in a 4-mm ssNMR rotor. Peptides were added to a final concentration of 2.4 mM (peptide–lipid ratio 1:25).

### 2.9. NMR Acquisition and Processing

The NMR assignment of mutants in solution was performed at 278 K. Complete assignment of amide, non-exchanging protons, and protonated ^13^C atoms was achieved in solution by ^1^H, ^13^C-HSQC, ^1^H, ^1^H-TOCSY (mixing of 60 ms), and ^1^H, ^1^H-NOESY (mixing of 200 ms) recorded on a 500 MHz (11.74 T) Bruker Avance DRX ultrashield spectrometer equipped with a 5 mm BBI (Broadband Inverse) probe. Typically, 16 and 64 scans were used with 32768 and 2048 × 256 points for 1D and 2D spectra, respectively. ^1^H, ^13^C-HSQC were acquired with 128 scans and 2048 × 512 points. A spectral window of 110 ppm was used centered at 50 ppm in the ^13^C dimension. In all cases, linear prediction was used in the indirect dimension. Relaxation delays were 2 s. In the presence of micelles, complete backbone assignment of Hα (and Cα when observable) was obtained similarly. Deuterated sodium TSP-d4 at a concentration of 100 µM was used as an internal reference for chemical shift (set to −0.015 in ^1^H dimension and −0.12 in ^13^C dimension) [51]. Reference random coil values in our experimental conditions (T = 278 K, pH 6.6 and ionic strength 0.01 M) were calculated by POTENCI web server (https://st-protein02.chem.au.dk/potenci/ (accessed on 11 October 2021)) [52].

Static ^2^H NMR spectra were acquired by solid-echo pulse sequence [53] at 500 MHz (4 mm multinuclear CP-MAS probe) using 32768 scans, a 90° pulse of 7.2 μs, and a defocusing delay (d6) of 40 μs. Measurements were performed at room temperature. Processing included the elimination of fid points preceding the echo (*nsp* and *ls* Bruker Topspin commands), Fourier transformation with line broadening, zero-order phasing, and baseline correction. First-order phase correction was not applied. ^31^P NMR spectra were acquired with a size of fid (TD) of 16,384 points, a 90° pulse of 5.0 μs, and a spectral width between 80–250 ppm according to the experiment to be performed. No proton decoupling was used. Magic-angle spinning (MAS) experiments were acquired with a spinning of 10 kHz. TopSpin 4 (Bruker BioSpin, Billerica, MA, USA) was used to process and analyze NMR data.

### 2.10. CD Spectroscopy

CD spectra were obtained in the far-UV (260–195 nm) on a J-815 Jasco spectropolarimeter (Tokyo, Japan) at 37 °C, using a 10 mm path cell, with 2 accumulations for a 216.0 μg/mL sample in 10 mM sodium phosphate buffer, pH 6.6.

### 2.11. Molecular Dynamics Simulations

Three lipid bilayer systems (POPC, POPS, POPC/POPS 50%:50%), in the presence and absence of peptides, were studied. Systems for simulations were prepared using “Membrane Builder” from CHARMM-GUI [54,55,56]. A total of 128 lipid molecules were placed in each lipid bilayer (i.e., 64 lipids in each leaflet) and peptide molecules were placed over the upper leaflet at non-interacting distance (>10 Å). Lysine residues were protonated. Initial peptide structure was obtained with I-TASSER [57,58,59]. Amidation of the C-terminus was achieved via the CHARMM terminal group patching functionality, integrated in CHARMM-GUI. A water layer of 50-Å thickness was added above and below the lipid bilayer which resulted in about 15000 water molecules with small variations depending on the nature of the membrane. Systems were neutralized with Na⁺ or Cl⁻ counterions.

MD simulations were performed using the GROMACS software [60] and CHARMM36m force field [61] under semi-isotropic NPT conditions [62,63]. The TIP3P model [64] was used to describe water molecules. Each system was energy-minimized with a steepest-descent algorithm for 5000 steps. Systems were equilibrated with the Berendsen barostat [65] and Parrinello–Rahman barostat [66,67] was used to maintain pressure (1 bar) semi-isotropically with a time constant of 5 ps and compressibility of 4.5 × 10^–5^ bar^−1^. The Nose–Hoover thermostat [68,69] was chosen to maintain the systems at 310 K with a time constant of 1 ps. All bonds were constrained using the LINear Constraint Solver (LINCS) algorithm, which allowed an integration step of 2 fs. Periodic boundary conditions (PBC) were employed for all simulations, and the particle mesh Ewald (PME) method [70] was used for long-range electrostatic interactions. After the standard CHARMM-GUI minimization and equilibration steps [62], the production run was performed for 500 ns. The whole process (minimization, equilibration, and production run) was repeated once more in the absence of peptide and twice more in its presence. Convergence was assessed using RMSD and polar contacts analysis.

All MD trajectories were analyzed using GROMACS tools [71,72]. MOLMOL [73] and VMD [74] were used for visualization. Graphs and images were produced with GNUplot [75] and PyMol [76].

## 3. Results and Discussion

### 3.1. Design of Ad-Hoc Mutations in Conserved Motifs Found in the HB43-Related Family of Anticancer Peptides

The family created by sequence alignment of anticancer peptides in the ADAPTABLE web server [46] was analyzed in terms of amino acid composition (Figure S1). The data showed that K and L are prevalently present, followed by I, G, A, F, and V. On average 3 K and 2 L are found in each peptide. In the family, anticancer peptides are composed of 4 basic (K/R), 6 hydrophobic, 2 aromatic, and 2 polar amino acids. In these sequences, lysine tends to be found after hydrophobic residues such as F, A, or L and to be followed by another lysine. RR motifs can also be found.

Among the ACPs, we selected HB43 (also called FLAK50) for its wide spectrum of activity toward different kinds of cancers: breast, colon, skin, lung, and cervix cancer tissues according to the database, CancerPPD (http://crdd.osdd.net/raghava/cancerppd/ (accessed on January 2019)) [8]. Its versatility suggests that its mechanism of action is not related to a specific receptor but involves general traits of cancer cells, as also suggested by our previous studies [42].

**Figure 1:**
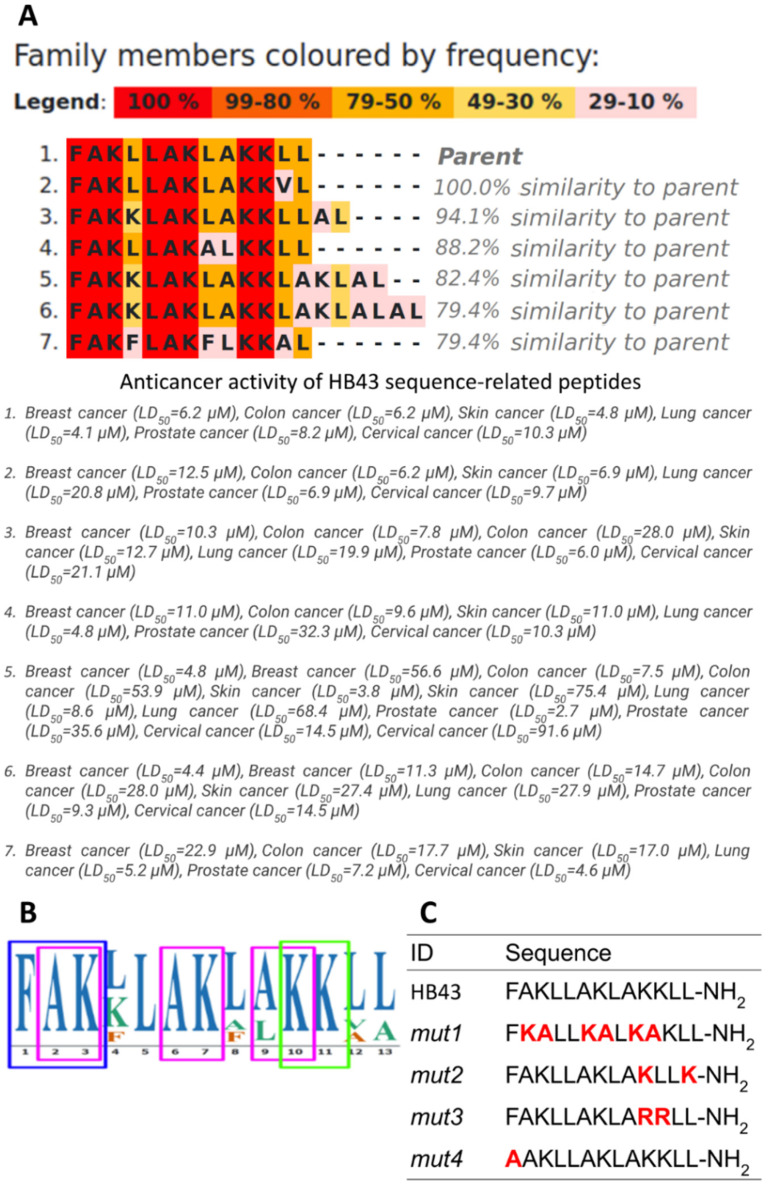
HB43-related family of anticancer peptides generated by the ADAPTABLE web server and anticancer activity of its members. (**A**) Sequence alignment highlighted conserved short motifs that could be at the origin of the activity. (**B**) ADAPTABLE-generated consensus sequence highlighting the conserved motifs. (**C**) Sequences of HB43 mutants, designed to alter putative motifs. Alterations are highlighted in red.

Using HB43 (FAKLLAKLAKKLL) as a template, we have created its related family of ACPs. Sequence alignment highlighted conserved short motifs that could be at the origin of the activity (Figure 1A). In particular, we identified the AK motif at different positions in the sequence, but also the KK motif with a 100% frequency (Figure 1B). All peptides also display FAK, LAK, and KK motifs. Interestingly K residues reappear cyclically every 4 amino acids, which suggests the periodicity of a helix (3.6 amino acids).

In order to highlight structure-function relations between these motifs and the activity, we intentionally perturbed the conserved parts of the sequence. Four new peptides were designed (Figure 1C): in *mut1* we have consistently inverted the AK motif; in *mut2* we altered the KK motif by separating the two Ks without changing the global composition in aminoacid types; in *mut3* the KK motif was replaced by RR to ascertain if the interaction with negatively charged cancer cell membranes is purely electrostatic or requires the functional groups of lysine residue; in *mut4* we substitute the N-terminal F by an A to investigate the role of the conserved aromatic residues which were proposed to have a role in other anticancer peptides such as aurein 1.2 [77,78].

**Figure 2:**
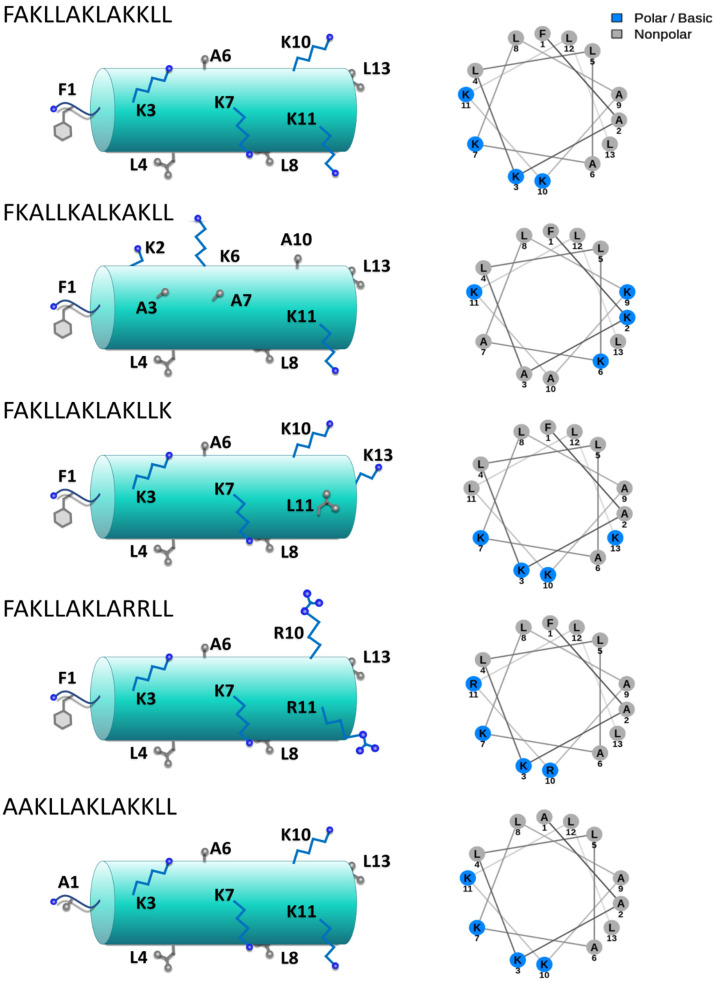
Schematic representation of HB43 and mutants (**left**) and their respective helical-wheel projections representing alpha-helix structures (**right**). Hydrophobic amino acids are represented in gray and those with a positive charge (K, R) in blue. Diagrams were created with NetWheels [82].

It should be noted that at variance with *mut1* and *mut2*, *mut3* and *mut4* do not alter the amphipathic character of a hypothetical alpha-helix which has been found for HB43 when interacting with membrane models [42] (Figure 2). Regarding *mut3*, it is worth mentioning that the guanidinium group of arginine was shown to bind to the phosphate oxygen of phospholipids in a multidentate fashion, thus possibly improving the penetration into target membranes [79,80], although this might result in toxicity [81].

### 3.2. Effect of HB43 and the Designed Mutants on Cancer Cell Viability

**Figure 3:**
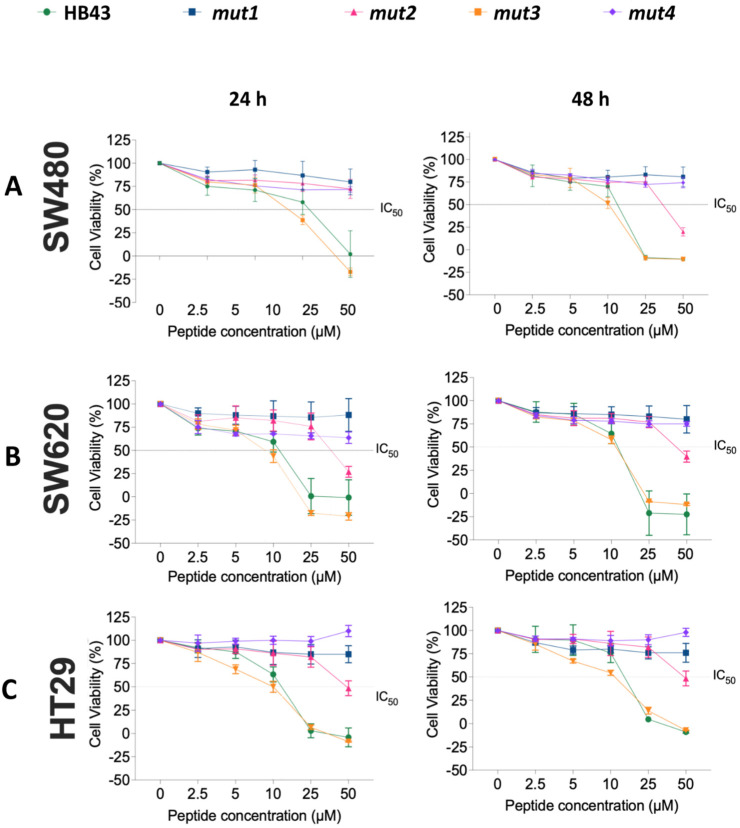
Effect of HB43 and mutants on cell viability of (**A**) SW480, (**B**) SW620, and (**C**) HT29 cells. Cells were treated with peptides for 24 h (**left**) and 48 h (**right**). Cell viability was measured using the AlamarBlue assay. Results were calculated as percent of control and represented as means ± S.D. of three independent experiments.

The effect of HB43 and its mutants were tested on human colon cancer (HT29, SW480, and SW620) cell lines. These are colorectal adenocarcinoma cell lines with epithelial morphology but different invasive capacities. Cells were treated with increasing concentrations (2.5 μM to 50 μM) of each peptide for 24 h and 48 h (Figure 3) and the half-maximal inhibitory concentration (IC_50_) was estimated (Table 1). HB43 showed a significant effect on cell viability after both 24 h and 48 h of treatment with all cell lines (the effect on HT29 cells is slightly less pronounced). *mut2* did not show any significant inhibitory effect, even if an abrupt reduction in cell viability was observed at the highest concentration (50 μM). No inhibitory effect on cell viability was detected when treating HT29, SW480, and SW620 cells with *mut1* and *mut4*. The most promising effect was observed for *mut3* with SW480 and SW620 cells, whose activity was slightly more cytotoxic than HB43. In this case, up to 50% reduction in cell viability was observed compared with untreated cells after 24 h of treatment at 10 μM peptide concentration, and ~40% after 48 h. This means that the replacement of two lysines by two arginines at positions 11–12 in the sequence of HB43 creates a peptide with similar or possibly more potent activity toward cancer cells.

In order to verify if our findings were specific to colon cancer cell lines or reflect a more general mechanism for cancer cells, the viability of breast cancer cells (MDA-MB231) was measured in the presence of increasing concentrations of all mutants (10 μM and 20 μM, Figure S2). Furthermore, in this case, *mut3* and HB43 display the highest anticancer activity, followed by *mut2*, while mut1 and *mut4* were not active. The results confirm our hypothesis, showing that very slight modifications of the sequence produce significant changes in the anticancer properties.

Even though most ACPs act by disrupting cancer cell membranes leading to cancer cell death, other mechanisms may act in synergy to enhance the anticancer activity [1,83,84]. These may include both extra and intracellular phenomena such as the inhibition of MMPs (metalloproteinases, enzymes involved in the degradation of the extracellular matrix) [85,86] or the perturbation of the cell cycle inhibiting proliferation [87].

### 3.3. HB43 and Mutants Do Not Alter Cell Cycle Distribution of Colon Cancer Cell Lines

Measuring the DNA content is an important strategy to unravel key factors in future clinical applications of anticancer agents since unregulated cell cycles are at the origin of processes such as the uncontrolled proliferation of cancer cells, induction of cell cycle arrest, and programmed death [88]. By means of fluorescent dyes, flow cytometry can monitor cellular DNA content, which in turn depends on the cell phase. This information can be used to (i) reveal the distribution of cells in different cycle phases (G0/G1 vs S vs G2/M), (ii) estimate the frequency of apoptotic cells with fractional DNA, and (iii) disclose DNA ploidy of a cell population [87].

The cell cycle was studied by flow cytometry (FL-2) on cancer cells HT29, SW480, and SW620 in the absence and in the presence of the most active peptides (HB43, *mut2*, and *mut3*). As shown in Figure S3, the cell cycle distribution of all cancer cell lines was unaffected after 12 h of treatment with 5 µM (HB43, *mut3*) and 20 µM (*mut2*). These results indicate that the peptides cannot alter the progression of the cell cycle in any of the cell lines under study.

### 3.4. HB43 and Mutants Do Not Have a Significant Impact on MMP Activity

Tumor invasion and metastasis are often accompanied by the degradation of the extracellular matrix (ECM). Invasiveness of cancer cells directly correlates with their ability to digest the elements of the ECM by the action of MMPs and thus their capability to metastasize [89,90]. Here, we tested whether the three most active peptides (HB43, *mut2*, and *mut3*) were capable of suppressing metalloproteinase (MMP) activity. Gelatin zymogram assay was used for the detection of MMP-2 and MMP-9 activity in the absence and in the presence of each peptide. No significant changes were observed (data not shown), although a slight decrease in MMP-9 activity was detected for HT-29 cells in all cases.

In helical conformation, HB43 displays an amphipathic structure (see Figure 2), typical of most antimicrobial peptides acting on biological membranes. Furthermore, our biological tests did not show a clear intracellular influence on the cell cycle. For these reasons, we decided to investigate the interaction with membrane models by NMR and MD simulations, to elucidate the mechanism of action at the molecular level of HB43 and its mutants. A comparison of structural data with the measured anticancer activity is then used to consolidate our model. To achieve this aim, we selected three peptides based on their activity: the most active (*mut3*), our reference HB43, and the rather inactive *mut4*.

### 3.5. NMR Assignment and Structure Determination of Mutants in Solution

**Figure 4:**
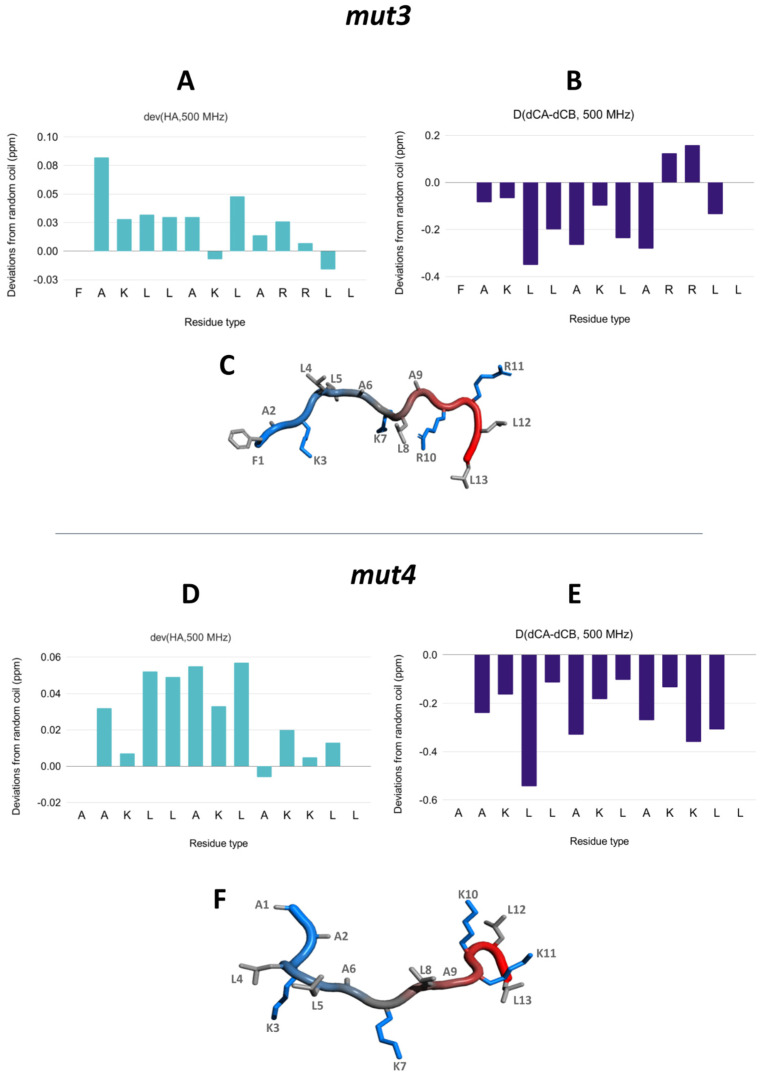
HB43 mutants tend to be unstructured in solution. (**A**,**B**,**D**,**E**) Chemical shift deviations from random coil values of Hα protons (**A**,**D**) and Cα–Cβ carbons (**B**,**E**) suggest the absence of the structure of *mut3* (top) and *mut4* (bottom). (**C**,**F**) Schematic representations of *mut3* (**C**) and *mut4* (**F**) are shown as a ‘tube’ colored from blue (N-terminus) to red (C-terminus). Sidechains are shown as sticks with the following color code: positively charged (blue) and nonpolar (light gray). The structures were created with PyMol [76]. Data on HB43 peptide were previously published [42].

The ^1^H and ^13^C chemical shift assignments of HB43 mutants are reported in Tables S1–S4. As for the case of HB43 peptide [42], NMR data are consistent with the absence of a well-defined structure. This is demonstrated by (i) the limited chemical shift dispersion resulting in severe overlap among resonances of the same residue types, (ii) the large linewidth of amide protons which improves by lowering the temperature (experiments were performed at 278 K), probably due to the reduced exchange rate with the solvent, (iii) the chemical shift index (CSI) performed on Hα, Cα and Cβ atoms (Figure 4) and (iv) CD spectra (Figure S5).

### 3.6. Interaction with Model Membranes

Peptide–lipid interactions with membrane models of cancer cells with increasing complexity were characterized by a combination of NMR and MD simulations.

#### 3.6.1. Structural Studies in Micelles

DPC micelles have been used as a very simplified model of biological membranes [91,92,93,94]. Even if they do not fully reproduce a phospholipid bilayer, they provide a hydrophobic environment with which the peptide can potentially interact. Moreover, the choline headgroup well reproduces phosphatidylcholine, the most abundant phospholipid in biological membranes.

**Figure 5:**
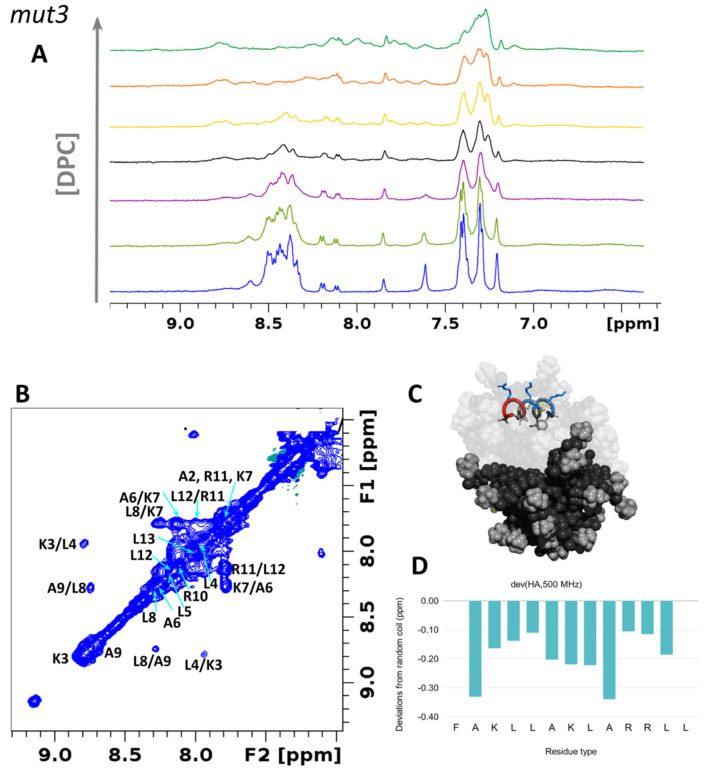
*mut3* assumes alpha-helical conformation in a lipidic environment. (**A**) ^1^H-NMR spectra of *mut3* 0.8 mM in the presence of DPC at concentrations of 2, 4, 8, 12, 24, and 50 mM. (**B**) ^1^H, ^1^H-NOESY spectrum of *mut3* at 278 K in the presence of DPC micelles showing meaningful NOEs in the amide region. (**C**) MD snapshot of *mut3* interacting with DPC micelles. The image was created with PyMol [76]. (**D**) Chemical shift deviations from random coil values of Hα hydrogen atoms. Data on HB43 peptide were previously published [42].

**Figure 6:**
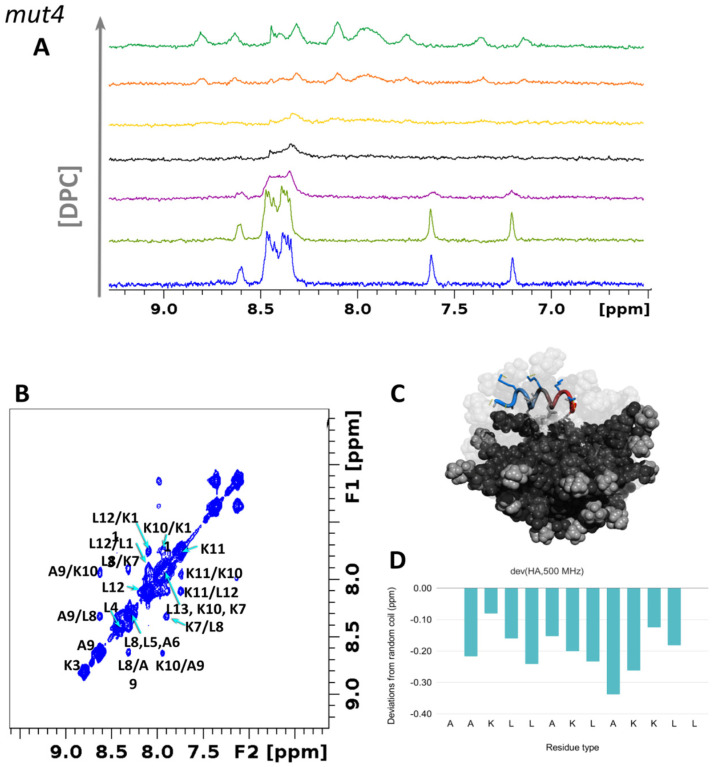
*mut4* assumes alpha-helical conformation in a lipidic environment. (**A**) ^1^H-NMR spectra of *mut4* 0.8 mM in the presence of DPC at concentrations of 2, 4, 8, 12, 24, and 50 mM. (**B**) ^1^H, ^1^H-NOESY spectrum of *mut4* at 278 K in the presence of DPC micelles showing meaningful NOEs in the amide region. (**C**) MD snapshot of *mut4* interacting with DPC micelles. The image was created with PyMol [76]. (**D**) Chemical shift deviations from random coil values of Hα hydrogen atoms. Data on HB43 peptide were previously published [42].

The addition of a concentrated solution of dodecylphosphatidyl-choline (DPC:d38) to a peptide sample dramatically affects the NMR spectrum in all cases (Figure 5A and Figure 6A). Amide protons from the unbound peptide shift and almost disappear but re-emerge as the DPC/peptide ratio increases (up to 60:1). Most importantly HN-HN cross-peaks appear in the NOESY spectrum (Figure 5B and Figure 6B), indicating the stabilization of alpha-helical conformations. Structuring of the peptide induced by the presence of DPC micelles (Figure 5C and Figure 6C) is confirmed by negative deviations of Hα protons’ chemical shifts with respect to their random coil values [52,95,96,97] (Figure 5D,E and Figure 6D,E). MD simulations further support what is observed by NMR and show the formation of salt bridges between the phosphate moieties of DPC and the amine of the N-terminus or lysine or arginine side chains (see polar contacts in Figure S4).

A closer analysis of the ^1^H,^13^C-HSQC spectrum reveals that the aromatic residue, Phe, is deeply affected by the presence of micelles in both HB43 and *mut3*. Interestingly, the new signals seem to be doubled as if the peptide form can bind in two different fashions. The large shift observed (about 0.16 ppm at 500 MHz) is compatible with the insertion of the aromatic ring in the bilayer (shielding effect) that would act as an anchoring point. The absence of such residue in *mut4* might result in a weaker binding with consequent weaker biological action.

#### 3.6.2. Interaction with DMPC/DHPC Bicelles

Isotropic bicelles, composed of the short-chain phospholipid diheptanoyl-sn-glycero-3-phosphocholine (DHPC) and the long-chain phospholipid dimyristoyl-sn-glycero-3-phosphocholine (DMPC), are able to form fast tumbling almost spherical bilayers amenable to liquid state NMR studies. The presence of a bilayer makes them a better model than micelles and they were used as membrane mimics to study the interaction of the HB43 mutants *mut3* and *mut4* with biological bilayers.

As in the case of micelles, the ^1^H-NMR spectra drastically change in the presence of isotropic bicelles (100 mM), reproducing the same effects observed in the presence of micelles. Amide and aromatic protons shift and almost disappear but re-emerge at different chemical shifts as the bicelle concentration is increased, suggesting the structuring of the peptides. The formation of an alpha-helical conformation is suggested by the appearance of HN-HN cross-peaks in the NOESY spectra (Figure S6A,B), and negative deviations of Hα protons (Cα signals are lost in the ^1^H,^13^C-HSQC spectrum due to faster T2 relaxation). Such an effect was observed with all peptides studied (*mut3*, *mut4,* and HB43) indicating that even the inactive peptide *mut4* is able to interact with bicelles.

Finally, as in the case of micelles, the ^1^H,^13^C-HSQC spectrum of HB43 and *mut3* reveal that the signals of the Phe1 aromatic sidechain shift considerably, suggesting the insertion of the aromatic ring into the bilayer, a phenomenon that cannot be observed for *mut4* which lacks such residue. A deep insertion of the aromatic ring was definitively confirmed by NOEs between the aromatic protons and the DHPC/DMPC lipid acyl chain (Figure S6C,D), clearly positioning the aromatic ring well inside the bilayer. Indeed, it has been shown that other aromatic moieties such as those of tryptophan and phenylalanine anchor to the polar–apolar interface [98,99,100,101].

#### 3.6.3. Interaction with SUVs

Small unilamellar vesicles (SUVs) are relatively good models of biological membranes. Compared to micelles, their curvature better represents that of a cell membrane. Moreover, the composition of phospholipids can be changed rather freely, allowing the modelization of different types of membranes. The relatively small dimensions of the SUVs (20–80 μm in diameter) and the fast dynamics of their phospholipids allow the detection of lipid signals under liquid NMR conditions [102]. On the other hand, proton detection of peptides interacting with SUVs depends on their degrees of freedom and is not always possible due to short T2 relaxation.

POPC/POPS SUVs were used to simulate the membrane of cancer cells, as exposure of PS on apoptotic cells is used as an “eat me” signal [103] for the identification of apoptotic and cancer cells by macrophages. One-dimensional ^1^H-NMR spectra of POPC/POPS SUVs were studied in the absence and in the presence of HB43, *mut3,* and *mut4*. In all cases, the peaks of the peptide are broad beyond detection indicating interaction. Despite the high lipid/peptide ratio, new broad signals appear in the aromatic regions, ascribable to the aromatic ring of the peptides (only HB43 and *mut3* contain aromatic residues). Such signals are observed only at temperatures as high as 310 K, probably due to the low mobility of the peptide at lower temperatures. Their large linewidths further testify to an interaction of HB43 and *mut3* peptides with SUV membrane models. As for *mut4*, severe overlap in the aliphatic region does not allow a clear detection of the peptide signals.

CD spectra nicely confirm our NMR results in micelles and bicelles, showing that also in the presence of SUV, all peptides assume alpha-helical conformation upon interaction with liposomes (Figure S5). Interestingly, in the case of inactive *mut4*, the transition is smoother indicating a weaker interaction.

#### 3.6.4. Interaction with MLVs

To evaluate the behavior of the phospholipid head groups, we conducted static and MAS ^31^P-NMR experiments of liposomes (a mixture of POPC/POPS 1:1) as a very basic model of cancerous eukaryotic cells.

The static ^31^P spectra show a broad signal typical shape of phospholipid liposomes with an isotropic peak corresponding to the phosphate buffer. The latter was used to identify free phosphate in MAS ^31^P-NMR experiments shown in Figure S7. In the absence of peptides, two main peaks are found corresponding to POPC and POPS which were assigned based on their relative intensity and previous literature [104,105]. The presence of all three peptides tends to affect the signal of POPS (Figure S7). However, when a larger amount of HB43 is added, both POPS and POPC are affected suggesting that the peptide interacts with phosphate moieties of both phospholipids.

### 3.7. MD Simulations of PeptideLipid Interactions

**Figure 7:**
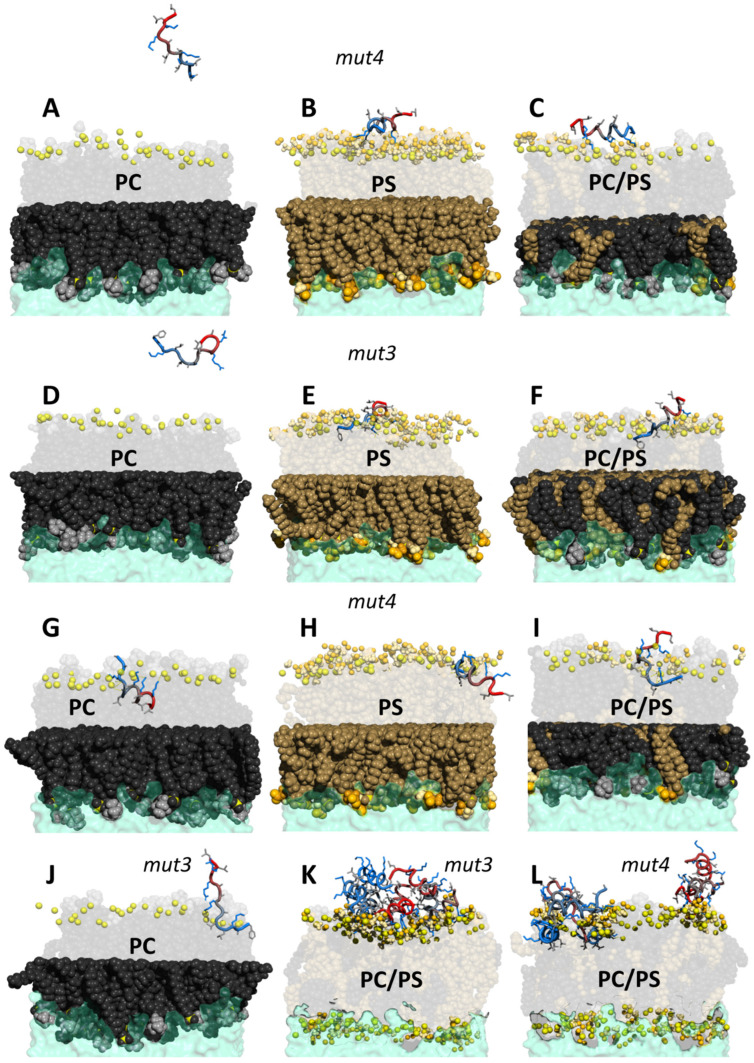
MD snapshots of *mut3* and *mut4* interacting with model membranes of various phospholipid compositions (**A**–**F**). Color code: phosphorus atom: yellow, POPC black (body) and light gray (choline group), POPS brown (body), gold (headgroup), light yellow (amine of the headgroup), and orange (carboxyl of the headgroup). For clarity, only functional groups of headgroups are shown (spheres) in the upper leaflet. *mut3* and *mut4* are shown as a ‘tube’ colored from blue (N-terminus) to red (C-terminus). Sidechains are shown as sticks with the following color code: positively charged (blue) and nonpolar (light gray). Snapshots in panels (**G**–**L**) refer to rare events observed along the trajectories. Data on HB43 peptide were previously published [42].

In order to get insight into the molecular interaction triggering the association of HB43 mutants with the membranes, we performed all-atom MD simulations with POPC/POPS mixtures as well as their pure components (Figure 7). Indeed, data reproduce qualitatively what is observed in biological tests, supporting the hypothesis of a mechanism of action based on the interaction with membranes.

The inactive *mut4* is attracted by the negatively charged PS containing membranes but tends to reside at the very surface (Figure 7A–C) while the active mutant *mut3* is able to penetrate PS containing bilayers (Figure 7D–F). It should be noted that what we describe is the most frequent overall behavior. Indeed, along with the three repetitions of 500 ns trajectories, we can find events where both *mut3* and *mut4* are able to penetrate all kinds of membranes (Figure 7G–J). This was observed also for HB43 [42] and it explains its weak hemolytic properties. However, both mutants are mostly found distant from pure PC membranes, here used to model non-cancerous cells. The different affinity of both mutants for PC and PS exposing membranes is clearly demonstrated by the area per lipid (Figure S8). The electrostatic attraction of both positively charged mutants exerts a pressure causing a reduction in the area of the external leaflet and a consequent increase in the area of the internal one.

The differences in behavior between *mut3* and *mut4* seem again to be related to the presence of the aromatic ring of phenylalanine. The occurrence of polar contacts along the trajectory can be monitored by calculating the radial distribution function [64] of each peptide polar atom from each phospholipid polar atom and extracting its maximum in the distance range of H-bonds and salt bridges. As observed in our experiments with bicelles, its ring is deeply inserted in the bilayer, a phenomenon confirmed by our simulations. Phenylalanine serves as an anchoring point linking *mut3* to the membrane. Two interactions contribute to synergy: a salt bridge between the N-terminal amine and the phosphate moiety of phospholipids and a van der Waals interaction between the aromatic ring and the lipid acyl chains (see polar and apolar contacts in Figure S9). Indeed, the importance of the FAK motif has been previously identified as crucial for the anticancer activity of HB43 [42]. Another anchoring point is provided by the establishment of salt bridges between the carboxylate oxygen of the serine headgroup with the guanidine of the arginine side chains (which were not present in HB43). This interaction is so strong that the peptide penetrates with the N-terminus but is not completely internalized (see Figure 7J), as in the case of HB43 [42], during the time length of our simulations. It should be noted that such salt bridges are established with the phosphate moieties rather than the serine headgroup in regions where the peptide (*mut3* or *mut4*) gets internalized.

Our calculations were repeated in the presence of eight peptides to simulate high peptide concentration and study possible inter-peptide interactions. Inter-residue contacts (Figure S10) clearly show that both mutants tend to aggregate on the surface (see Figure 7K,L), a phenomenon already discussed for HB43 [42] which is compatible with a carpet model mechanism of action.

## 4. Conclusions

ACPs are short peptides mainly composed of K and L amino acids (I, G, A, F, and V are also frequent) able to interact and disrupt the lipid architecture of cancer cell membranes. Taking the HB43 as a reference for its vast anticancer activity, we identified conserved stretches of residues (motifs) and showed that the activity is dramatically changed by both changing the order or nature of their amino acids. Mutations altering the amphipathic structure of the helix formed by HB43 on cancer cells’ mimetic liposomes [42], generally result in a weaker activity while those leaving such structure unaffected may result in a loss of activity (e.g., substitution of F with A in *mut4*) or an enhancement (e.g., substitution of KK motif for RR). This, together with the absence of effect on the cell cycle and inhibition of MMPs, further support that our ACPs act at the level of the cell membrane. Having ascertained the importance of maintaining the amphipathic structure, we performed NMR and MD studies on *mut3* (the peptide with enhanced activity) and *mut4* (the A1F mutant with no activity). We found that both get structured forming an alpha-helix in the presence of lipidic systems mimicking cancer cell membranes but interact poorly with PC membranes representing non-cancerous eukaryotic cells. However, in the case of *mut3*, the presence of phenylalanine in the FAK motif allows the peptide to penetrate deeply with its N-terminus, as testified by the direct NOEs observed between the acyl chain of phospholipid and the aromatic ring of phenylalanine. Furthermore, MD simulations show that the guanidinium group of the two arginines replacing the KK motif efficiently binds to the carboxylate of PS, usually exposed on the surface of apoptotic cancer cells. When internalized the same groups can form salt bridges with the phosphate oxygen atoms of phospholipids thus explaining the additional antibacterial activity of both HB43 and its *mut3* mutant.

## Figures and Tables

**Table 1:** Half-maximal inhibitory concentration (IC_50_) of HB43, *mut2* and *mut3* peptides (μM) in colon cancer cell lines SW480, SW620, and HT29 after 24-h and 48-h treatment.

		24 h			48 h	
Peptide	SW480	SW620	HT29	SW480	SW620	HT29
HB43	12 ± 4	10 ± 1	11 ± 1	11.4 ± 0.3	10.9 ± 0.7	13.3 ± 0.4
mut2	34 ± 2	40 ± 3	50 ± 3	39 ± 3	47 ± 3	50 ± 3
mut3	8 ± 1	9 ± 1	9 ± 1	10.0 ± 0.4	10.94 ± 0.07	9.5 ± 0.5

Data are expressed as IC_50_ and are means ± S.D. of three independent experiments.

## Data Availability

Not applicable.

## Supplementary Materials

The following supporting information can be downloaded at: https://www.mdpi.com/article/10.3390/pharmaceutics14051089/s1, Figure S1: Analysis of the amino acid composition of HB43-related family generated by ADAPTABLE web server, Figure S2: Cell viability of MDA-MB 231 breast cancer cells treated with HB43 and mutants, Figure S3: Analysis of cell percentages in each cell cycle phase for colon cancer cells under study, HT29 (**left**), SW480 (**center**), and SW620 (**right**). Cells were treated with peptides HB43/*mut3* (5 µM), *mut2* (20 µM) and incubated for 12 h. Each data point is expressed as mean ± SD (n = 2), Table S1: ^1^H and ^13^C NMR assignment of *mut3* 0.8 mM in 10 mM phosphate buffer pH 6.6, 10% D_2_O, 278 K, Table S2: ^1^H NMR assignment of *mut3* 0.8 mM (90% 10 mM phosphate buffer at pH 6.6, 10% D_2_O) in the presence of 50 mM DPC micelles at 278 K, Table S3: ^1^H and ^13^C NMR assignment of *mut4* 0.8 mM in 10 mM phosphate buffer pH 6.6, 10% D_2_O, 278 K, Table S4: ^1^H NMR assignment of *mut4* 0.8 mM (90% 10 mM phosphate buffer at pH 6.6, 10% D_2_O) in the presence of 50 mM DPC micelles at 278 K, Figure S4: Occurrence of polar atom contacts (H-bonds and salt bridges) (**A**) and van der Waals contacts (**B**) between *mut3* and *mut4* with DPC micelles calculated along MD simulation trajectories, Figure S5: CD spectra of HB43 (**A**), *mut3* (**B**), and *mut4* (**C**) 10 µM in 10 mM phosphate buffer at pH 6.6, in the absence (light green) and in the presence of increasing amounts of POPC/POPS SUVs (0, 6.67 × 10^6^, 1.33 × 10^5^, 2.00 × 10^5^, 3.33 × 10^5^, 4.67 × 10^5^, 6.00 × 10^5^, 7.33 × 10^5^, 8.67 × 10^5^, 1.00 × 10^4^, 1.13 × 10^4^, 1.27 × 10^4^, 1.40 × 10^4^, and 1.53 × 10^4^ M), Figure S6: Amide and aromatic regions of ^1^H,^1^H-NOESY NMR spectrum of *mut3* and *mut4* 1.6 mM in 10 mM phosphate buffer at pH 6.6 (blue) and 310 K, in the presence of DMPC/DHPC isotropic bicelles at a total lipid concentration of 100 mM. (**A**,**B**) Amide region of *mut3* (**A**) and *mut4* (**B**) showing meaningful NOEs. (**C**,**D**) side-chain spectral regions of *mut3* (**C**) where aromatic signals of phenylalanine (Phe1) clearly show cross-peaks with the lipid chains of bicelles, a phenomenon not observed for *mut4* (**D**) due to the absence of this residue, Figure S7: MAS ^31^P spectra of POPC/POPS (1:1) liposomes in the absence (blue) and the presence of HB43 (green), *mut3* (magenta), *mut4* (black), and a more concentrated sample of HB43 (yellow), Figure S8: Area per lipid (nm^2^) in bilayers containing POPC and POPS as calculated from MD simulations in the presence of eight peptides of *mut3* (**A**,**B**) and *mut4* (**C**,**D**). The average value is shown in blue, while the upper and lower leaflet are shown in yellow and red, respectively, Figure S9: Occurrence of polar atom contacts (H-bonds and salt bridges) and van der Waals contacts between *mut3* (**top**), *mut4* (**bottom**), and POPC/POPS bilayers calculated along MD simulation trajectories, Figure S10: Contact maps in simulated POPC/POPS systems when eight peptides of *mut3* (**A**) and *mut4* (**B)** are present, Figure S11: Analytical purity of mut1. HPLC C12 column (Phenomenex^®^ C12, Jupiter 4 µ Proteo, 90 Å, 250 × 4.6 mm) using a mixture of aqueous 0.1% (*v*/*v*) TFA (**A**) and 0.1% (*v*/*v*) TFA in acetonitrile (**B**) as the mobile phase (flow rate of 1 mL/min) and employing UV detection at 210 nm, Figure S12: Analytical purity of *mut2*. HPLC C12 column (Phenomenex^®^ C12, Jupiter 4 µ Proteo, 90 Å, 250 × 4.6 mm) using a mixture of aqueous 0.1% (*v*/*v*) TFA (**A**) and 0.1% (*v*/*v*) TFA in acetonitrile (**B**) as the mobile phase (flow rate of 1 mL/min) and employing UV detection at 210 nm, Figure S13: Analytical purity of *mut3*. HPLC C12 column (Phenomenex^®^ C12, Jupiter 4 µ Proteo, 90 Å, 250 × 4.6 mm) using a mixture of aqueous 0.1% (*v*/*v*) TFA (**A**) and 0.1% (*v*/*v*) TFA in acetonitrile (**B**) as the mobile phase (flow rate of 1 mL/min) and employing UV detection at 210 nm, Figure S14. Analytical purity of *mut4*. HPLC C12 column (Phenomenex^®^ C12, Jupiter 4 µ Proteo, 90 Å, 250 × 4.6 mm) using a mixture of aqueous 0.1% (*v*/*v*) TFA (A) and 0.1% (*v*/*v*) TFA in acetonitrile (**B**) as the mobile phase (flow rate of 1 mL/min) and employing UV detection at 210 nm.
