# Supplementary material for: The Influence of Short Motifs on the Anticancer Activity of HB43 Peptide
Source: Pharmaceutics. 2022 May 19;14(5):1089. doi: 10.3390/pharmaceutics14051089 (PMC9147034; doi:10.3390/pharmaceutics14051089)
Supplement: Supplementary file 1 [file pharmaceutics-14-01089-s001.zip › pharmaceutics-1707274-supplementary.pdf]

Supporting Information

The Influence of Short Motifs on the Anticancer Activity of HB43 Peptide

Claudia Herrera-León [1], Francisco Ramos-Martín [1], Hassan El Btaouri [2], Viviane Antonietti [3], Pascal Sonnet [3], Laurent Martiny [2], Fabrizia Zevolini [4], Chiara Falciani [4], Catherine Sarazin [1] and Nicola D'Amelio [1,*]

[1] Unité de Génie Enzymatique et Cellulaire UMR 7025 CNRS, Université de Picardie Jules Verne, 80039 Amiens, France; claudia.herrera@u-picardie.fr (C.H.-L.); francisco.ramos@u-picardie.fr (F.R.-M.); catherine.sarazin@u-picardie.fr (C.S.)

[2] Matrice Extracellulaire et Dynamique Cellulaire UMR 7369 CNRS, Université de Reims Champagne Ardenne (URCA), 51100 Reims, France; hassan.elbtaouri@univ-reims.fr (H.E.B.); laurent.martiny@univ-reims.fr (L.M.)

[3] Agents Infectieux, Résistance et Chimiothérapie, AGIR UR 4294, Université de Picardie Jules Verne, UFR de Pharmacie, 80037 Amiens, France; viviane.silva-pires@u-picardie.fr (V.A.); pascal.sonnet@u-picardie.fr (P.S.)

[4] Department of Medical Biotechnology, University of Siena, 53100 Siena, Italy; fabrizia.zevolini@student.unisi.it (F.Z.); chiara.falciani@unisi.it (C.F.)

* Correspondence: nicola.damelio@u-picardie.fr; Tel.: +33-3-22-82-74-73; Fax: +33-3-22-82-75

Motifs:
Position-independent probability to find a residue type at a certain distance from a reference residue type

e.g. if an Alanine (A) is present one peptide of the family, the graph relative to A reports on the probability to find each residue type n positions (or regions) apart.

(A)Alanine
(R)Arginine
(N)Asparagine
(D)Aspartate
(C)Cysteine
(Q)Glutamine
(E)Glutamate
(G)Glycine
(H)Histidine
(I)Isoleucine
(L)Leucine
(K)Lysine
(M)Methionine
(F)Phenylalanine
(P)Proline
(S)Serine
(T)Threonine
(W)Tryptofan
(Y)Tyrosine
(V)Valine

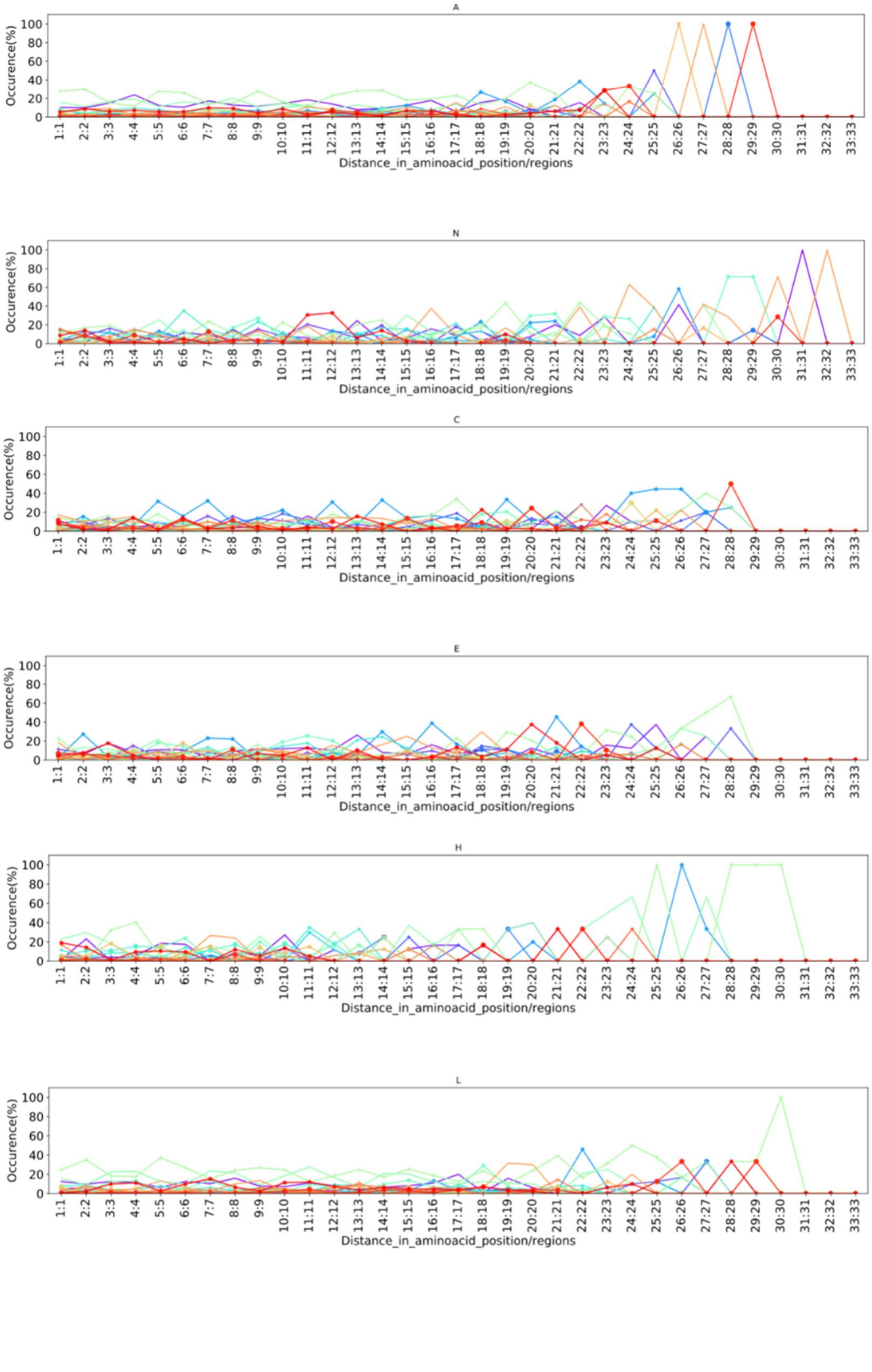

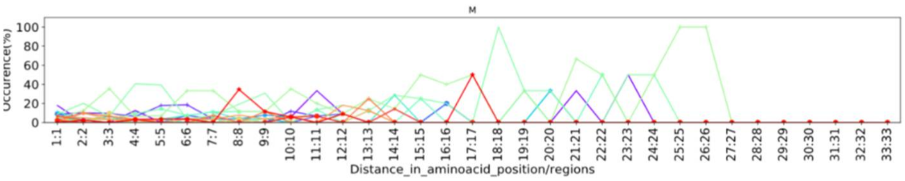
M
Occurence(%)
Distance_in_aminoacid_position/regions

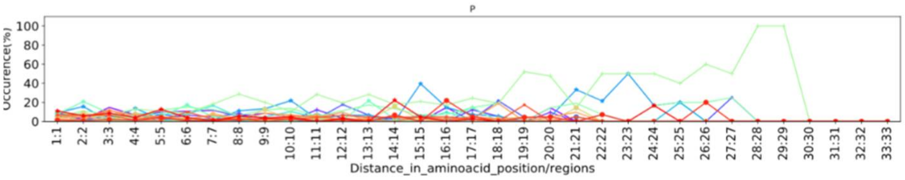
P
Occurence(%)
Distance_in_aminoacid_position/regions

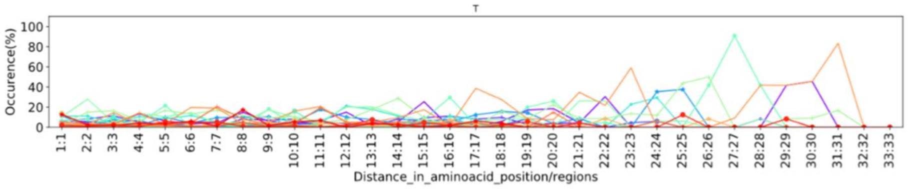
T
Occurence(%)
Distance_in_aminoacid_position/regions

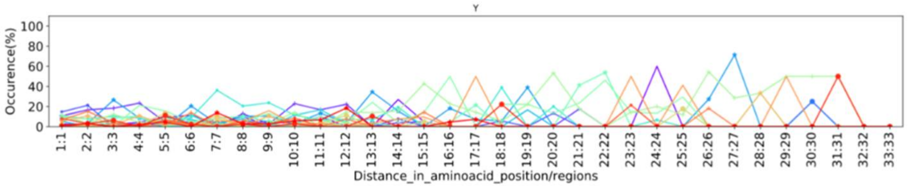
Y
Occurence(%)
Distance_in_aminoacid_position/regions

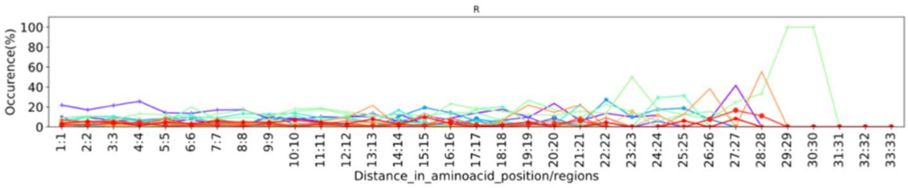
R
Occurence(%)
Distance_in_aminoacid_position/regions

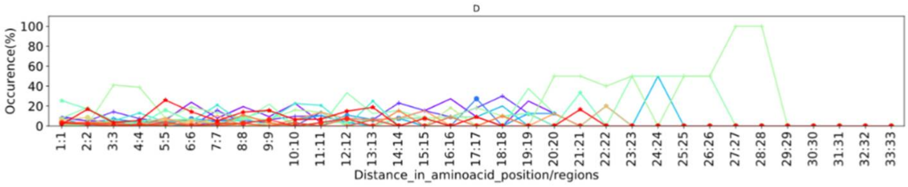
D
Occurence(%)
Distance_in_aminoacid_position/regions

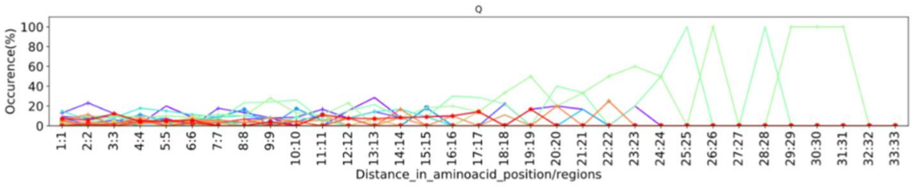
Q
Occurence(%)
Distance_in_aminoacid_position/regions

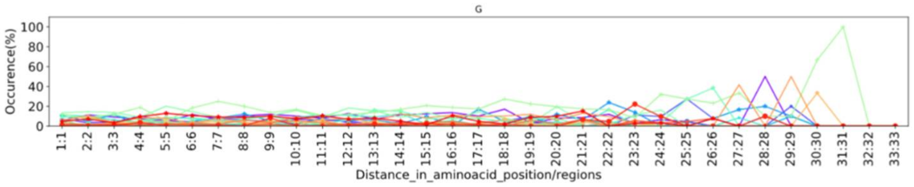
G
Occurence(%)
Distance_in_aminoacid_position/regions

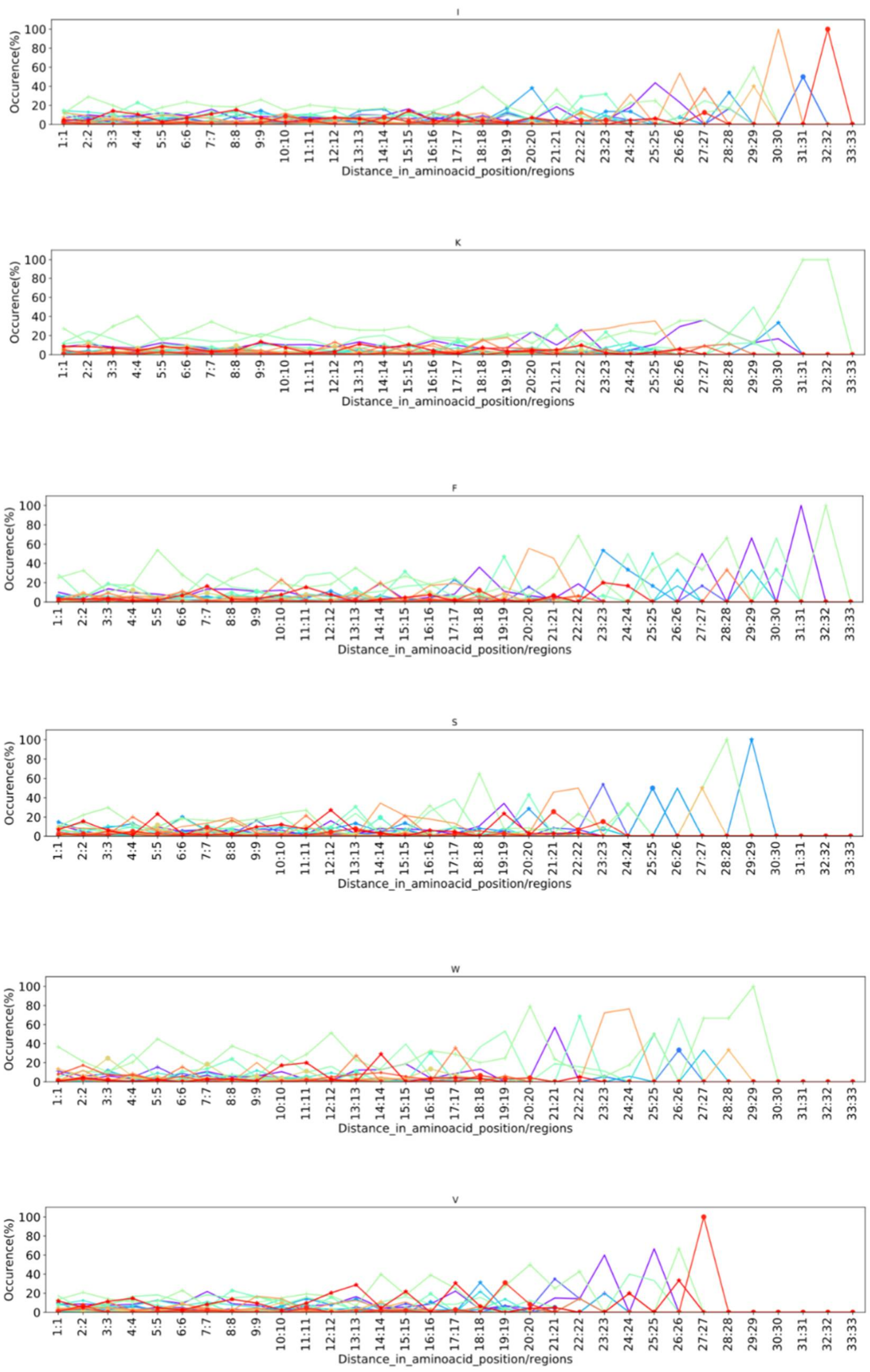

Figure S1. Analysis of the amino acid composition of HB43-related family generated by ADAPTABLE web server.

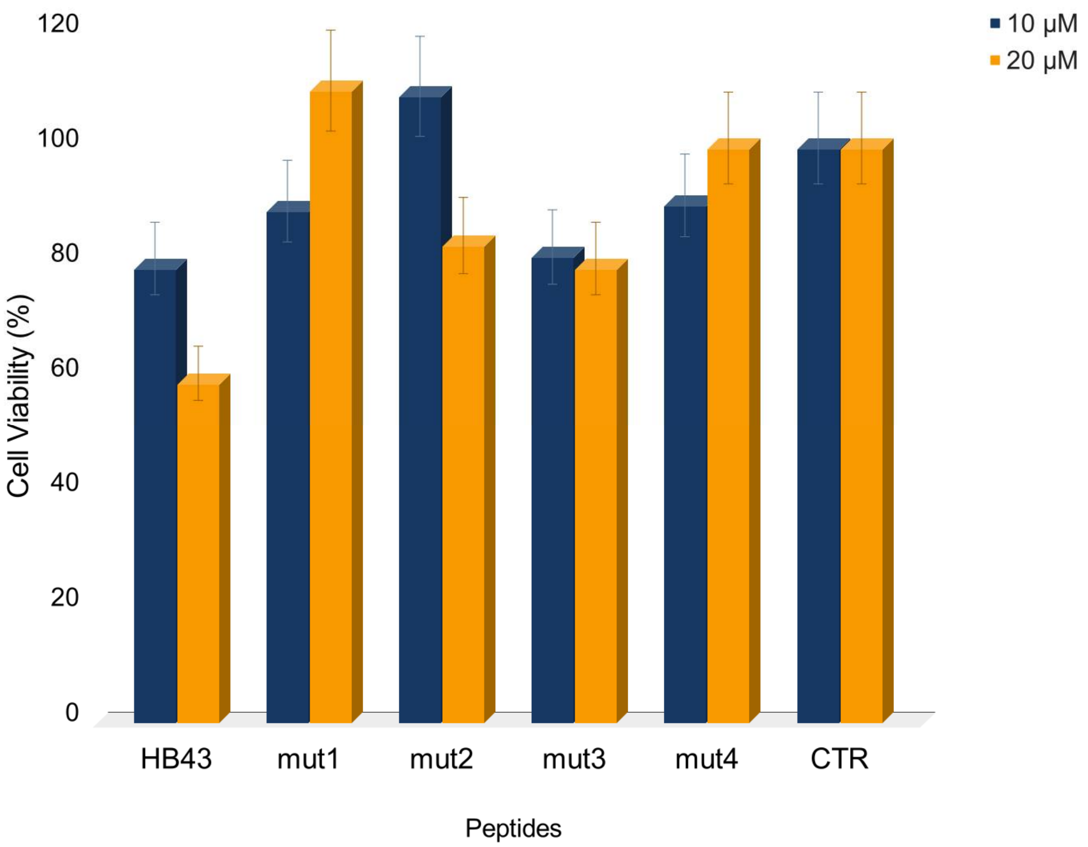

Figure S2. Cell viability of MDA-MB 231 breast cancer cells treated with HB43 and mutants.

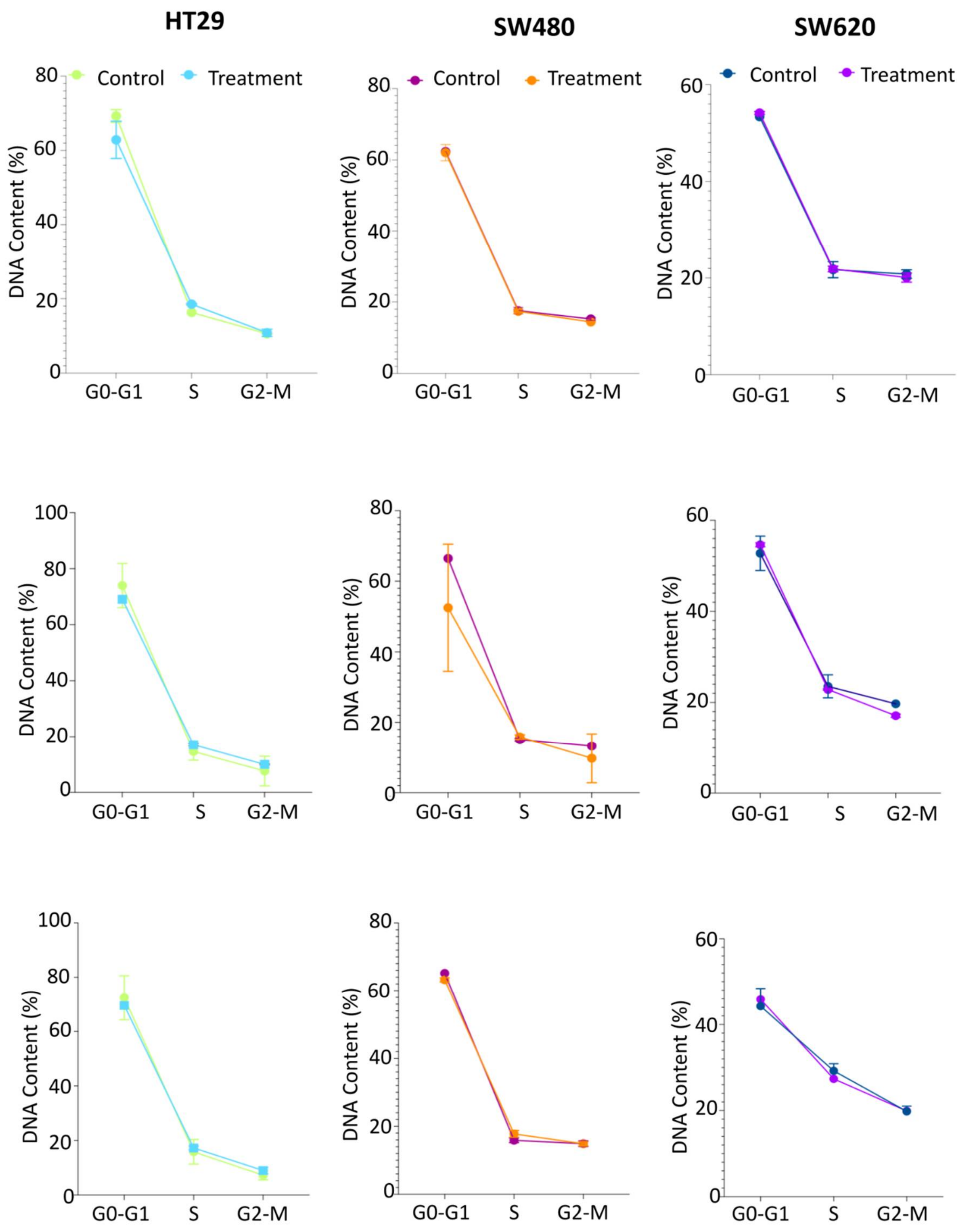

Figure S3. Analysis of cell percentages in each cell cycle phase for colon cancer cells under study, HT29 (**left**), SW480 (**center**), and SW620 (**right**). Cells were treated with peptides HB43/*mut3* (5 µM), *mut2* (20 µM) and incubated for 12 h. Each data point is expressed as mean ± SD (n = 2).

Table S1. ^{1}H and ^{13}C NMR assignment of *mut3* 0.8 mM in 10 mM phosphate buffer pH 6.6, 10% D_2O, 278 K

	**Phe 1**	**Ala 2**	**Lys 3**	**Leu 4**	**Leu 5**	**Ala 6**	**Lys 7**
^{1}H	NH=X	NH= 8.47	NH= 8.62	NH= 8.59	NH= 8.41	NH= 8.55	NH= 8.54
	α =4.19	α = 4.33	α = 4.24	α = 4.33	α = 4.33	α = 4.27	α = 4.24
	β_1 = 3.1	β = 1.37	β_1 = 1.79	β = 1.63	β = 1.63	β = 1.39	β_1 = 1.79
	β_2 = 3.25		β_2 = 1.79	γ = 1.66	γ = 1.66		β_2 = 1.79
	δ = 7.30		γ = 1.48	δ_1 = 0.96	δ_1 = 0.96		γ = 1.48
	ϵ = 7.41		δ = 1.71	δ_2 = 0.89	δ_2 = 0.89		δ = 1.71
	ζ = 7.38		ϵ = 3.00				ϵ = 3.00
^{13}C	α =57.22	α = 52.66	α = 56.64	α = 55.09	α = 55.09	α = 52.64	α = 56.64
	β = 40.2	β = 19.41	β = 33.23	β = 42.34	β = 42.34	β = 19.26	β = 33.23
	δ = 132.36		γ = 25.08	γ = 26.99	γ = 26.99		γ = 25.08
	ϵ = 131.98		δ = 29.35	δ_1 = 25.13	δ_1 = 25.13		δ = 29.35
	ζ = 130.78		ϵ = 42.11	δ_2 = 23.45	δ_2 = 23.45		ϵ = 42.11

	**Leu 8**	**Ala 9**	**Arg 10**	**Arg 11**	**Leu 12**	**Leu 13**	
^{1}H	NH= 8.51	NH= 8.72	NH= 8.50	NH= 8.60	NH= 8.61	NH= 8.43	
	α = 4.33	α = 4.33	α = 4.29	α = 4.29	α = 4.33	α = 4.33	
	β = 1.63	β = 1.39	β_2 = 1.81	β_2 = 1.81	β = 1.634	β = 1.63	
	γ = 1.66		β_3 = 1.81	β_3 = 1.81	γ = 1.66	γ = 1.66	
	δ_1 = 0.96		γ = 1.66	γ = 1.66	δ_1 = 0.96	δ 1 = 0.96	
	δ_2 = 0.89		δ = 3.21	δ = 3.21	δ_2 = 0.89	δ_2 = 0.89	
			NH_1 = 7.01	NH_1 = 7.68			
			NH_2 = 6.56	NH_2 = 6.27			
^{13}C	α = 55.09	α = 52.37	α = 56.45	α = 56.45	α = 55.09	α = 55.09	
	β = 42.34	β = 19.26	β = 30.82	β = 30.82	β = 42.34	β = 42.34	
	γ = 26.99		γ = 27.43	γ = 27.43	γ = 26.99	γ = 26.99	
	δ_1 = 25.13		δ = 43.45	δ = 43.45	δ_1 = 25.13	δ_1= 25.13	
	δ_2 = 23.45				δ_2 = 23.45	δ_2 = 23.45	

Table S2. ^{1}H NMR assignment of *mut3* 0.8 mM (90% 10 mM phosphate buffer at pH 6.6, 10% D_2O) in the presence of 50 mM DPC micelles at 278 K

Phe 1	Ala 2	Lys 3	Leu 4	Leu 5	Ala 6	Lys 7
NH=X	NH= 7.9	NH= 8.79	NH= 7.94	NH= 8.18	NH= 8.24	NH= 7.79
α =3.98	α = 3.92	α = 4.05	α = 4.16	α = 4.19	α = 4.04	α = 4.03
β_1 = 3.13	β = 1.43	β_1 = 1.85	β = 1.82	β = 1.72	β = 1.55	β_1 = 2.01
β_2 = 3.14		β_2 = 1.85	γ = 1.47	γ = 1.50		β_2 = 2.01
δ = 7.27		γ = 1.45	δ_1 = 0.91	δ_1 = 0.91		γ = 1.55
ϵ = 7.32		δ = 1.60	δ_2 = 0.91	δ_2 = 0.91		δ = 1.70
ζ = ?		ϵ = 3.30				ϵ = ?
**Leu 8**	**Ala 9**	**Arg 10**	**Arg 11**	**Leu 12**	**Leu 13**	
NH= 8.28	NH= 8.75	NH= 8.14	NH= 7.79	NH= 8.14	NH= 8.01	
α = 4.06	α = 3.92	α = 4.16	α = 4.17	α = 4.17	α = 4.14	
β = 1.79	β = 1.51	β_2 = ?	β_2 = ?	β = 1.86	β = 1.84	
γ = 1.49		β_3 = ?	β_3 = ?	γ = 1.51	γ = 1.55	
δ_1 = 0.92		γ = ?	γ = ?	δ_1 = 0.95	δ_1 = 0.92	
δ_2 = 0.97		δ = ?	δ = ?	δ_2 = 0.91	δ_2 = 0.92	
		NH_1 = ?	NH_1 = ?			
		NH_2 = ?	NH_2 = ?			

Missing values are due to overlap or exchange with the solvent (exchangeable protons). Severe broadening is observed for lysine and arginine side chains preventing their full assignment.

Table S3. ^1H and ^{13}C NMR assignment of *mut4* 0.8 mM in 10 mM phosphate buffer pH 6.6, 10% D_2O, 278 K

	**Ala 1**	**Ala 2**	**Lys 3**	**Leu 4**	**Leu 5**	**Ala 6**	**Lys 7**
^1H	NH= X	NH= 8.80	NH= 8.72	NH= 8.47	NH= 8.58	NH= 8.47	NH= 8.55
	α = 4.07	α = 4.31	α = 4.25	α = 4.35	α = 4.35	α = 4.30	α = 4.28
	β = 1.53	β = 1.39	β_1 = 1.78	β = 1.62	β = 1.62	β = 1.39	β_1 = 1.73
			β_2 = 1.78	γ = 1.66	γ = 1.66		β_2 = 1.73
			γ = 1.47	δ_1 = 0.95	δ_1 = 0.95		γ = 1.46
			δ = 1.70	δ_2 = 0.89	δ_2 = 0.89		δ = 1.70
			ϵ = 3.01				ϵ = 3.01
^{13}C	α = 51.79	α = 52.58	α = 56.73	α = 55.07	α = 55.35	α = 52.65	α = 56.62
	β = 19.72	β = 19.37	β = 33.43	β = 42.53	β = 42.53	β = 19.37	β = 33.31
			γ = 25.08	γ = 27.20	γ = 27.20		γ = 25.08
			δ = 29.42	δ_1 = 25.07	δ_1 = 25.07		δ = 29.42
			ϵ = 42.26	δ_2 = 23.66	δ_2 = 23.66		ϵ = 42.26

	**Leu 8**	**Ala 9**	**Lys 10**	**Lys 11**	**Leu 12**	**Leu 13**	
^1H	NH= 8.58	NH= 8.67	NH= 8.56	NH= 8.45	NH= 8.52	NH= 8.49	
	α = 4.35	α = 4.26	α = 4.26	α = 4.26	α = 4.36	α = 4.33	
	β = 1.62	β = 1.39	β_1 = 1.78	β_1 = 1.78	β = 1.62	β = 1.62	
	γ = 1.66		β_2 = 1.78	β_2 = 1.78	γ = 1.66	γ = 1.66	
	δ_1 = 0.95		γ = 1.47	γ = 1.47	δ_1 = 0.95	δ_1 = 0.95	
	δ_2 = 0.89		δ = 1.70	δ = 1.70	δ_2 = 0.89	δ_2 = 0.89	
			ϵ = 3.01	ϵ = 3.01			
^{13}C	α = 55.35	α = 52.65	α = 56.61	α = 56.49	α = 55.10	α = 55.21	
	β = 42.53	β = 19.37	β = 33.31	β = 33.43	β = 42.53	β = 42.53	
	γ = 27.204		γ = 25.08	γ = 25.08	γ = 27.20	γ = 27.20	
	δ_1 = 25.07		δ = 29.42	δ = 29.42	δ_1 = 25.07	δ_1 = 25.07	
	δ_2 = 23.66		ϵ = 42.26	ϵ = 42.26	δ_2 = 23.66	δ_2 = 23.66	

Table S4. ^1H NMR assignment of *mut4* 0.8 mM (90% 10 mM phosphate buffer at pH 6.6, 10% D_2O) in the presence of 50 mM DPC micelles at 278 K

**Ala 1**	**Ala 2**	**Lys 3**	**Leu 4**	**Leu 5**	**Ala 6**	**Lys 7**
NH= X	NH= 8.12	NH= 8.8	NH= 8.39	NH= 8.32	NH= 8.32	NH= 7.89
α = 4.13	α = 4.06	α = 4.16	α = 4.14	α = 4.06	α = 4.09	α = 4.05
β = 1.52	β = 1.53	β_1 = 1.91	β = 1.87	β = 1.85	β = 1.54	β_1 = 2.04
		β_2 = 1.91	γ = 1.68	γ = ?		β_2 = 2.04
		γ = 1.51	δ_1 = ?	δ_1 = 0.92		γ = 1.53
		δ = 1.71	δ_2 = ?	δ_2 = 0.92		δ = 1.70
		ϵ = ?				ϵ = ?
**Leu 8**	**Ala 9**	**Lys 10**	**Lys 11**	**Leu 12**	**Leu 13**	
NH= 8.32	NH= 8.63	NH= 7.95	NH= 7.75	NH= 8.09	NH= 7.99	
α = 4.06	α = 3.93	α = 3.97	α = 4.13	α = 4.16	α = 4.27	
β = 1.84	β = 1.50	β_1 = 1.95	β_1 = 2.02	β = 1.84	β = 1.82	
γ = ?		β_2 = 1.95	β_2 = 2.02	γ =?	γ = 1.82	
δ_1 = 0.92		γ = 1.52	γ = ?	δ_1 = 0.91	δ_1 = 0.92	
δ_2 = 0.92		δ = 1.72	δ = ?	δ_2 = 0.91	δ_2 = 0.92	
		ϵ = ?	ϵ =?			

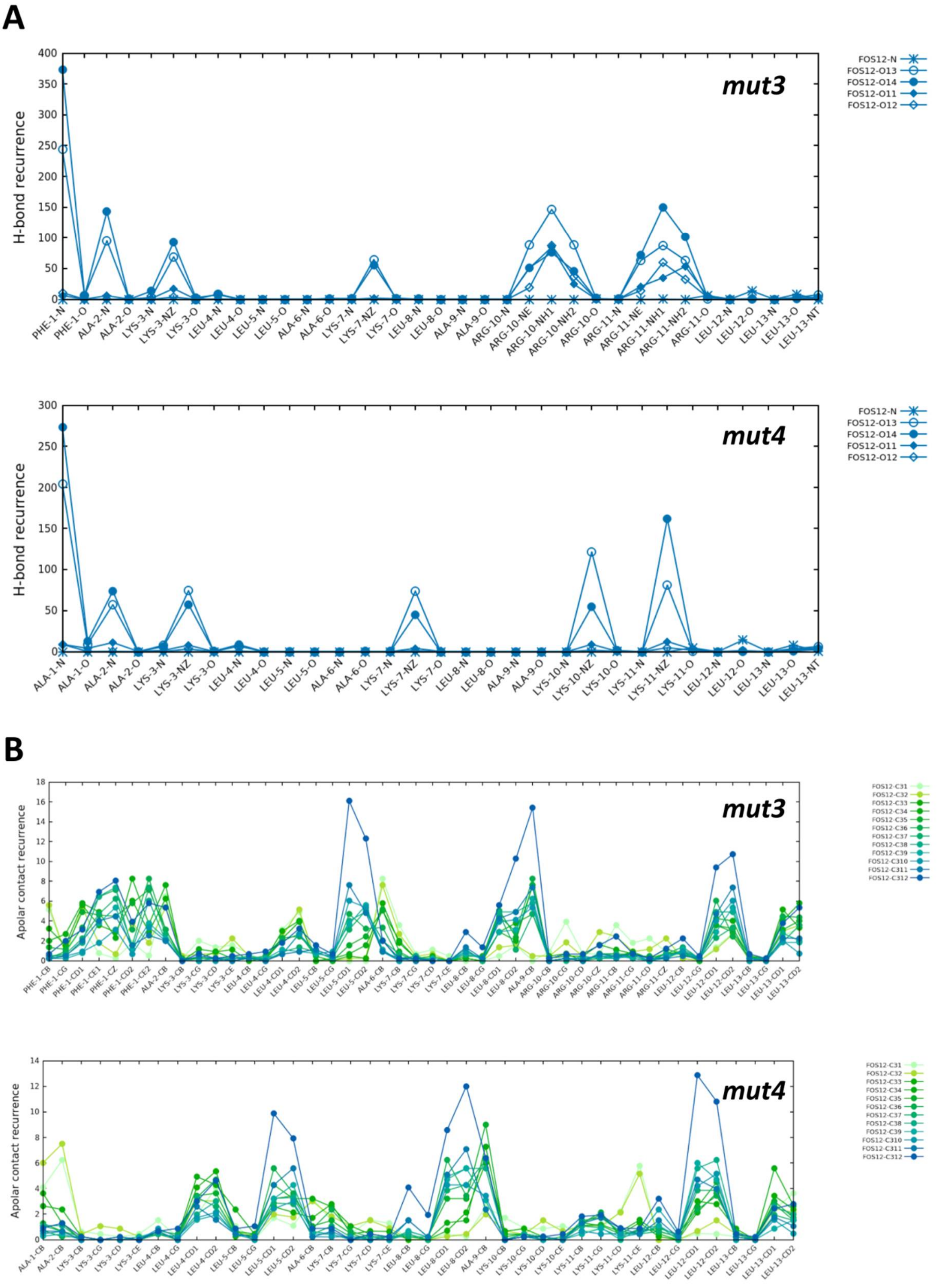

Figure S4. Occurrence of polar atom contacts (H-bonds and salt bridges) (**A**) and van der Waals contacts (**B**) between *mut3* and *mut4* with DPC micelles calculated along MD simulation trajectories.

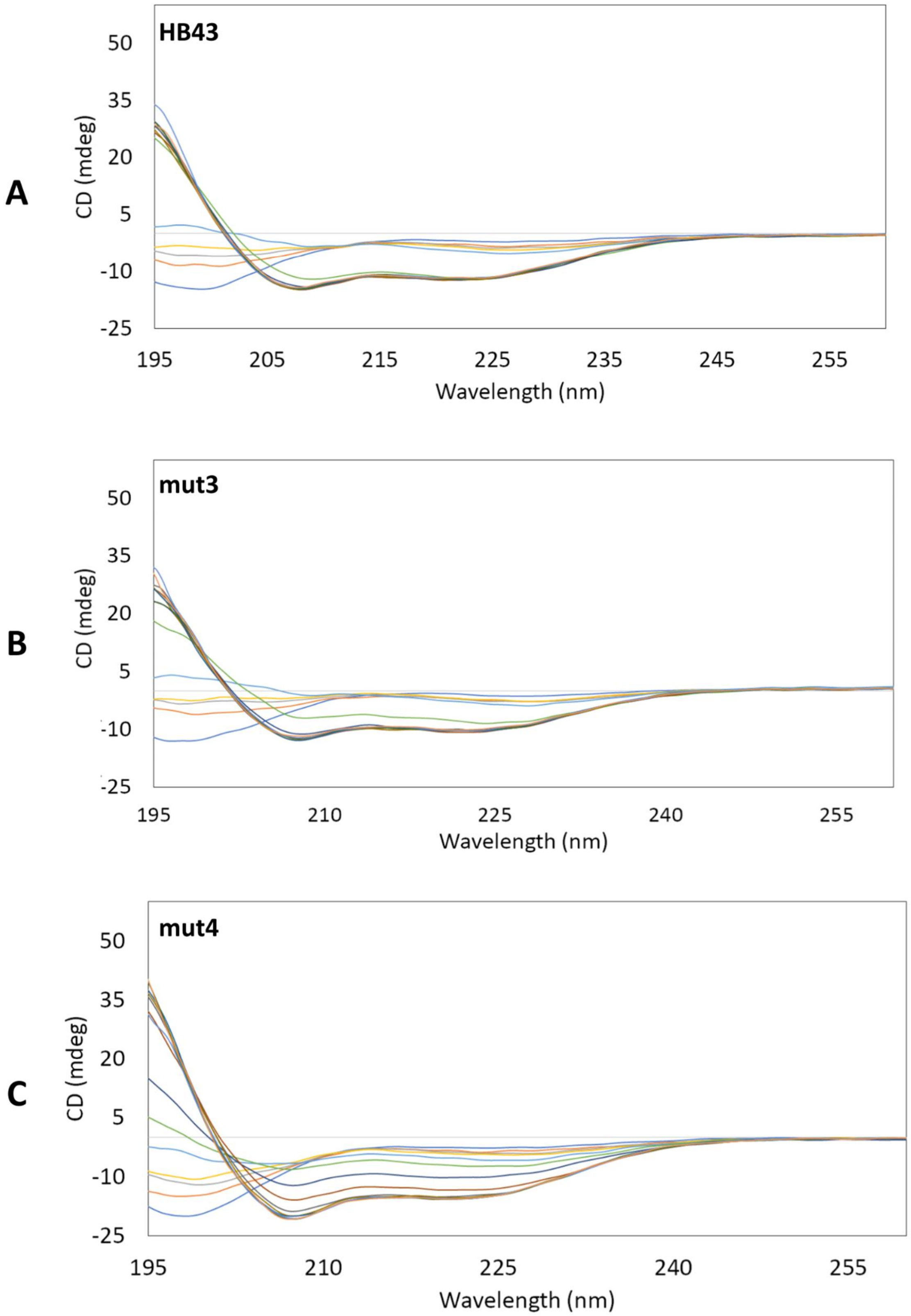

Figure S5. CD spectra of HB43 (**A**), *mut3* (**B**), and *mut4* (**C**) 10 μM in 10 mM phosphate buffer at pH 6.6, in the absence (light green) and in the presence of increasing amounts of POPC/POPS SUVs (0, 6.67×10^{6}, 1.33×10^{5}, 2.00×10^{5}, 3.33×10^{5}, 4.67×10^{5}, 6.00×10^{5}, 7.33×10^{5}, 8.67×10^{5}, 1.00×10^{4}, 1.13×10^{4}, 1.27×10^{4}, 1.40×10^{4}, and 1.53×10^{4} M).

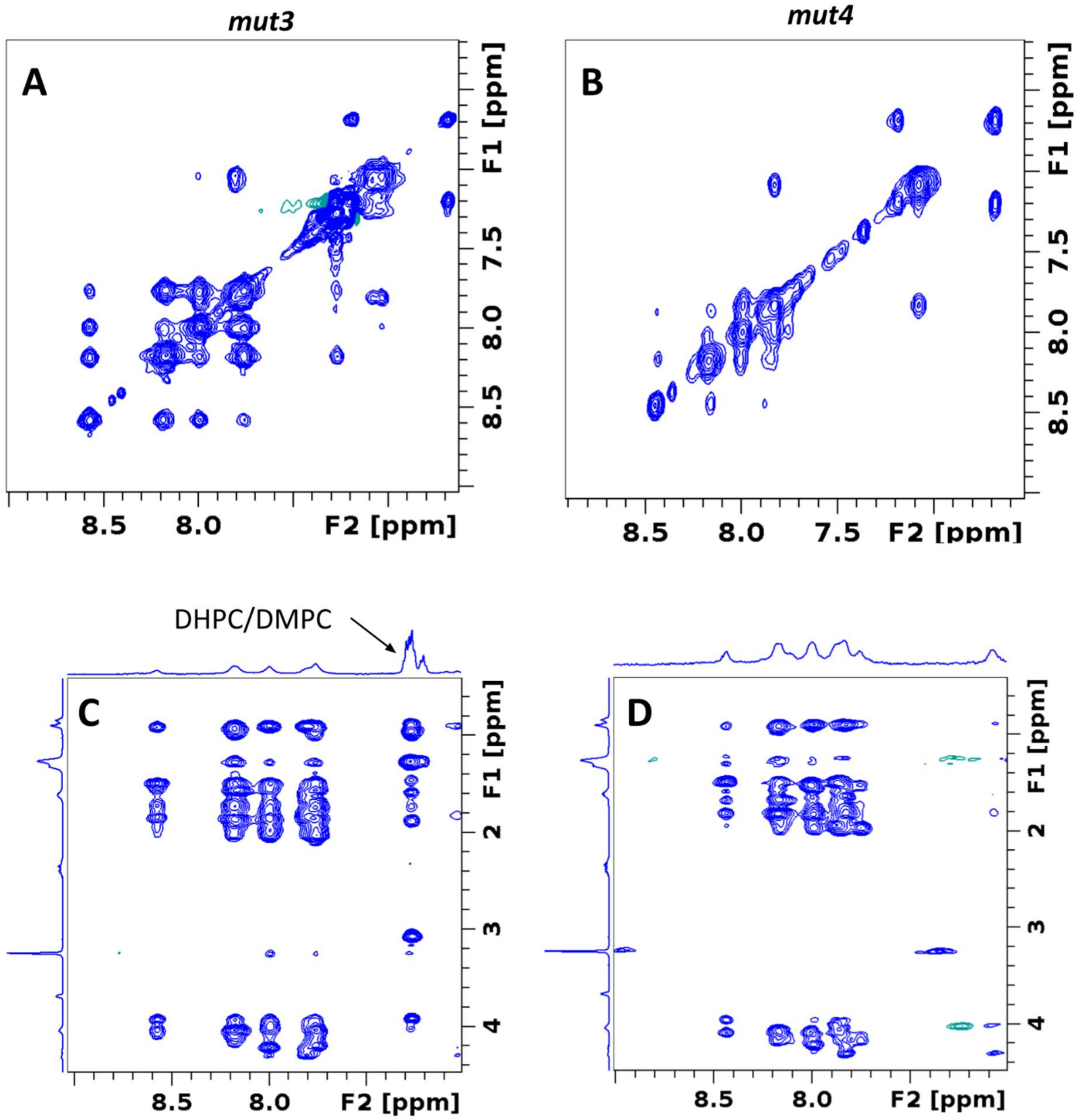

Figure S6. Amide and aromatic regions of $^{1}H,^{1}H$-NOESY NMR spectrum of *mut3* and *mut4* 1.6 mM in 10 mM phosphate buffer at pH 6.6 (blue) and 310 K, in the presence of DMPC/DHPC isotropic bicelles at a total lipid concentration of 100 mM. (**A**,**B**) Amide region of *mut3* (**A**) and *mut4* (**B**) showing meaningful NOEs. (**C**,**D**) side-chain spectral regions of *mut3* (**C**) where aromatic signals of phenylalanine (Phe1) clearly show cross-peaks with the lipid chains of bicelles, a phenomenon not observed for *mut4* (**D**) due to the absence of this residue.

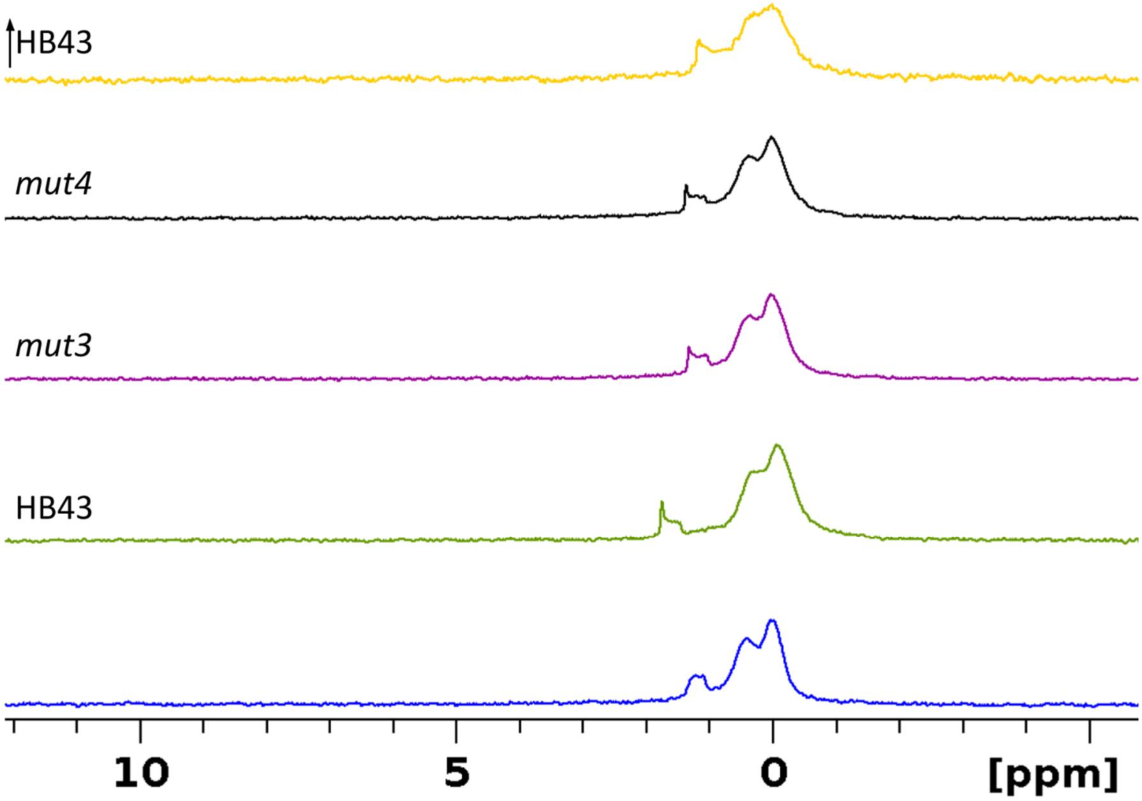

Figure S7. MAS ^{31}P spectra of POPC/POPS (1:1) liposomes in the absence (blue) and the presence of HB43 (green), *mut3* (magenta), *mut4* (black), and a more concentrated sample of HB43 (yellow).

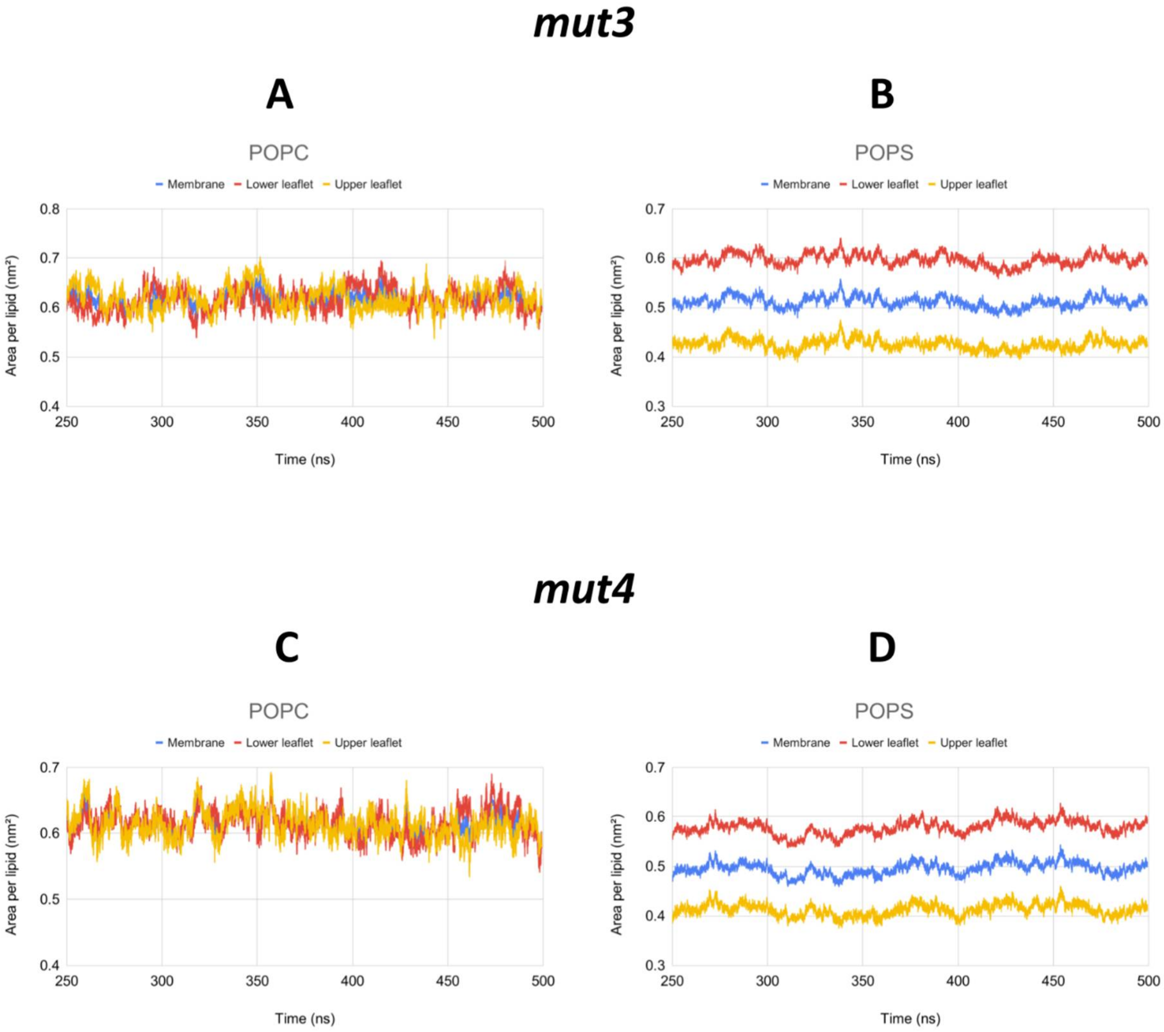

Figure S8. Area per lipid (nm^2) in bilayers containing POPC and POPS as calculated from MD simulations in the presence of eight peptides of *mut3* (**A**,**B**) and *mut4* (**C**,**D**). The average value is shown in blue, while the upper and lower leaflet are shown in yellow and red, respectively.

POPC/POPS

mut3

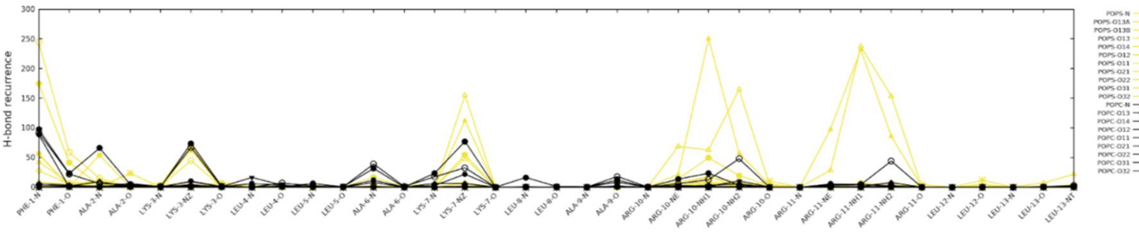

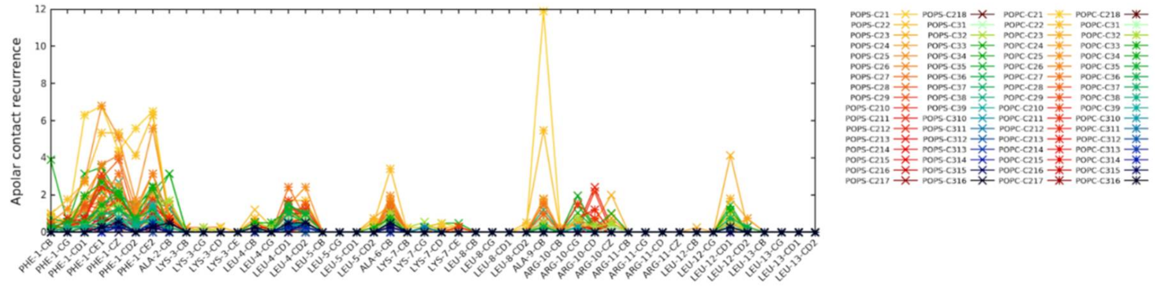

mut4

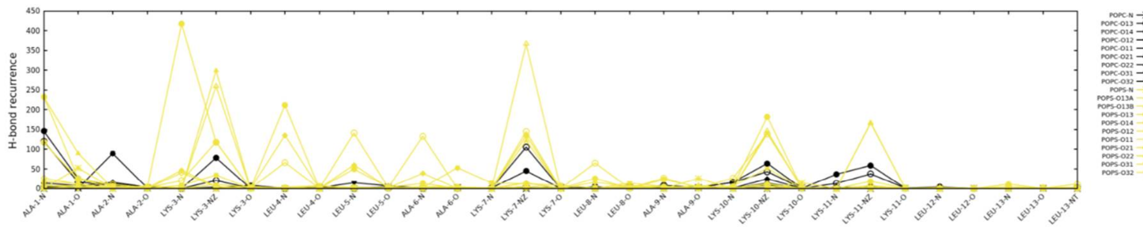

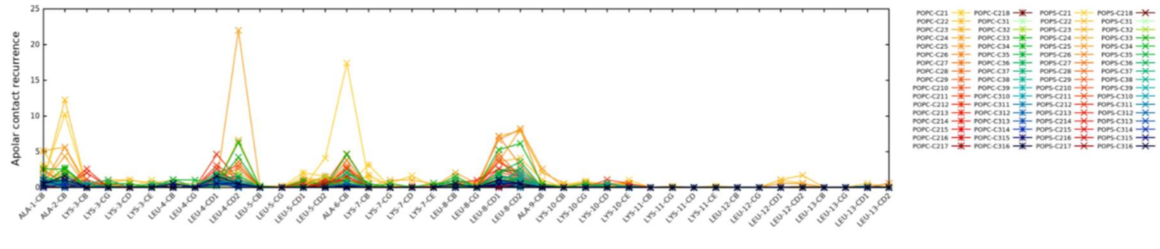

Figure S9. Occurrence of polar atom contacts (H-bonds and salt bridges) and van der Waals contacts between *mut3* (**top**), *mut4* (**bottom**), and POPC/POPS bilayers calculated along MD simulation trajectories.

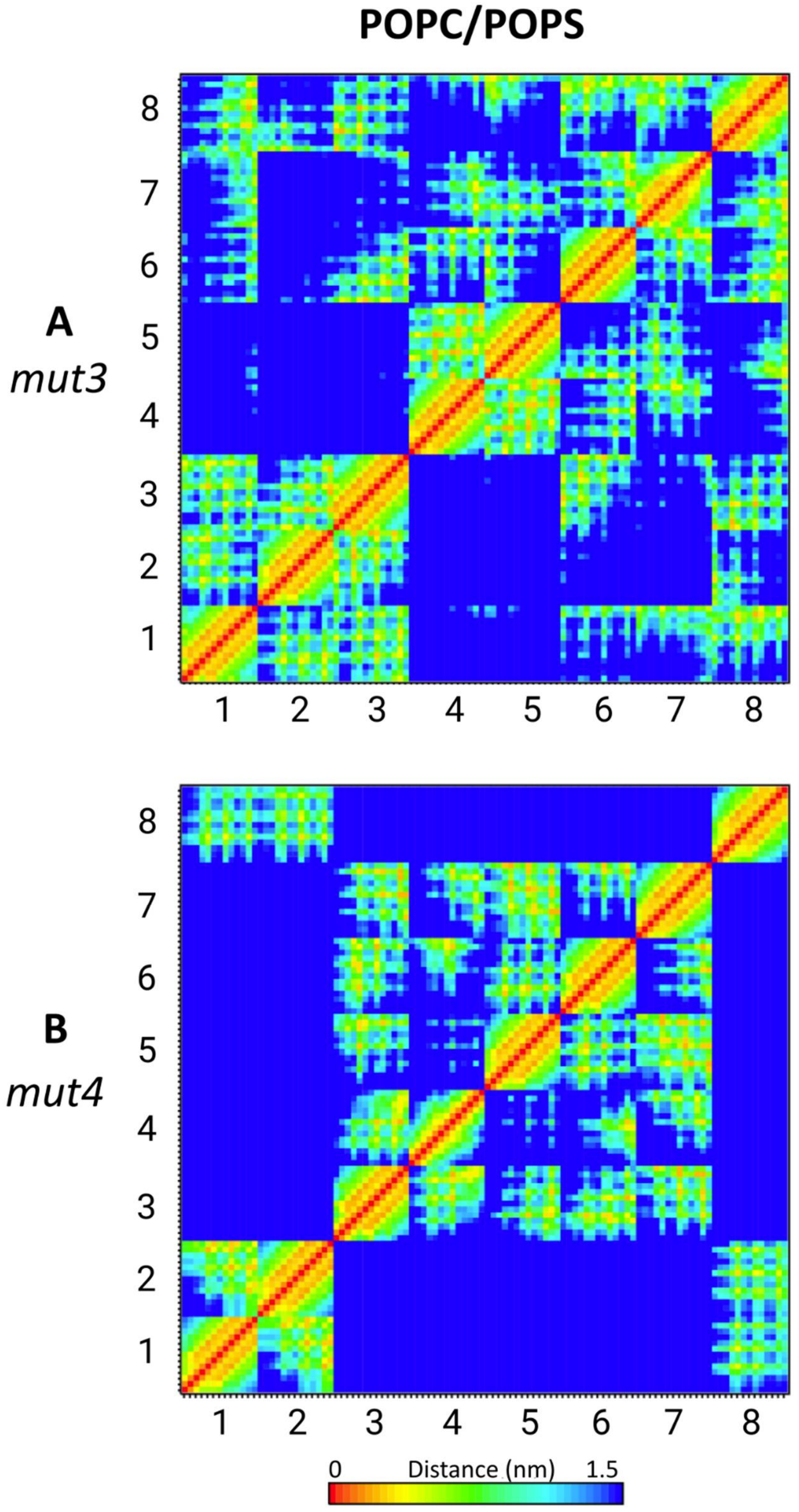

Figure S10. Contact maps in simulated POPC/POPS systems when eight peptides of *mut3* (**A**) and *mut4* (**B)** are present.

FKALLKALKAKLL-NH_2 (***mut1***)

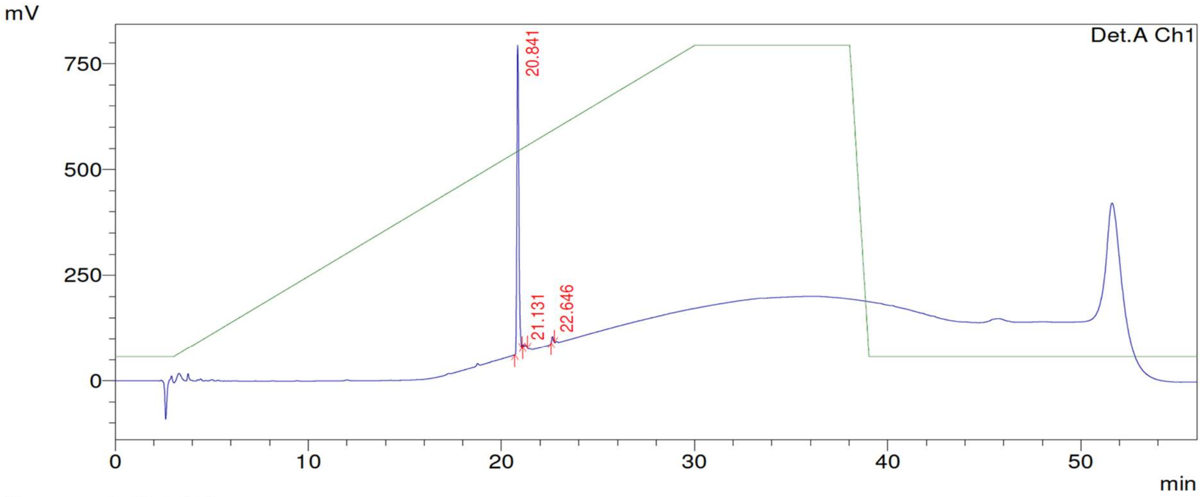

Detector A Ch1 210nm

Peak Purity Index	Ret. Time	Area	Height	Area %	Height %
	20.841	5065200	725793	97.472	97.346
	21.131	52512	5500	1.011	0.738
	22.646	78835	14284	1.517	1.916
		5196547	745577	100.000	100.000

Figure S11. Analytical purity of mut1. HPLC C12 column (Phenomenex® C12, Jupiter 4 µ Proteo, 90 Å, 250 × 4.6 mm) using a mixture of aqueous 0.1% (*v*/*v*) TFA (**A**) and 0.1% (*v*/*v*) TFA in acetonitrile (**B**) as the mobile phase (flow rate of 1 mL/min) and employing UV detection at 210 nm.

FAKLLAKLAKLLLK-NH_2 (***mut2***)

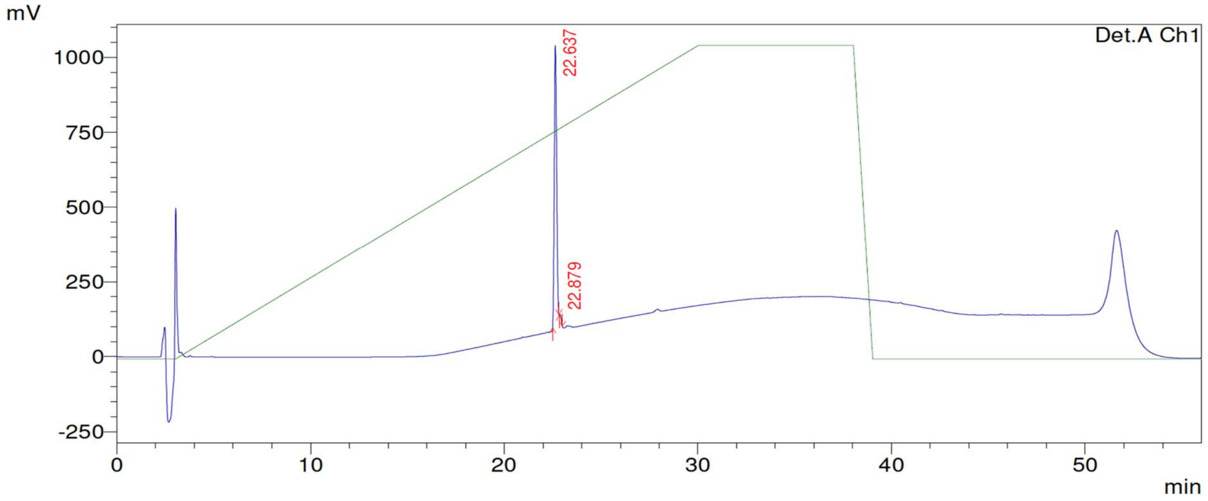

Detector A Ch1 210nm

Peak Purity Index	Ret. Time	Area	Height	Area %	Height %
	22.637	7822238	924470	99.316	98.977
	22.879	53894	9558	0.684	1.023
		7876132	934028	100.000	100.000

Figure S12. Analytical purity of *mut2*. HPLC C12 column (Phenomenex® C12, Jupiter 4 µ Proteo, 90 Å, 250 × 4.6 mm) using a mixture of aqueous 0.1% (*v*/*v*) TFA (**A**) and 0.1% (*v*/*v*) TFA in acetonitrile (**B**) as the mobile phase (flow rate of 1 mL/min) and employing UV detection at 210 nm.

FAKLLAKLARRLL-NH_2 (***mut3***)

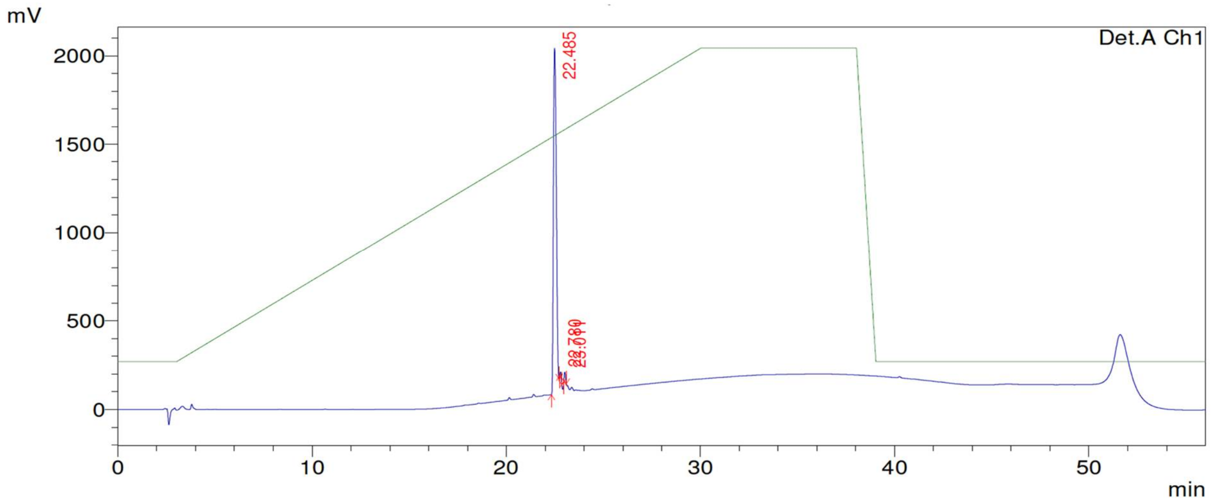

Detector A Ch1 210nm

Peak Purity Index	Ret. Time	Area	Height	Area %	Height %
	22.485	19782282	1922920	97.975	95.341
	22.780	140362	35185	0.695	1.745
	23.011	268462	58779	1.330	2.914
		20191106	2016884	100.000	100.000

Figure S13. Analytical purity of *mut3*. HPLC C12 column (Phenomenex® C12, Jupiter 4 µ Proteo, 90 Å, 250 × 4.6 mm) using a mixture of aqueous 0.1% (*v*/*v*) TFA (**A**) and 0.1% (*v*/*v*) TFA in acetonitrile (**B**) as the mobile phase (flow rate of 1 mL/min) and employing UV detection at 210 nm.

AAKLLAKLAKKLL-NH_2 (***mut4***)

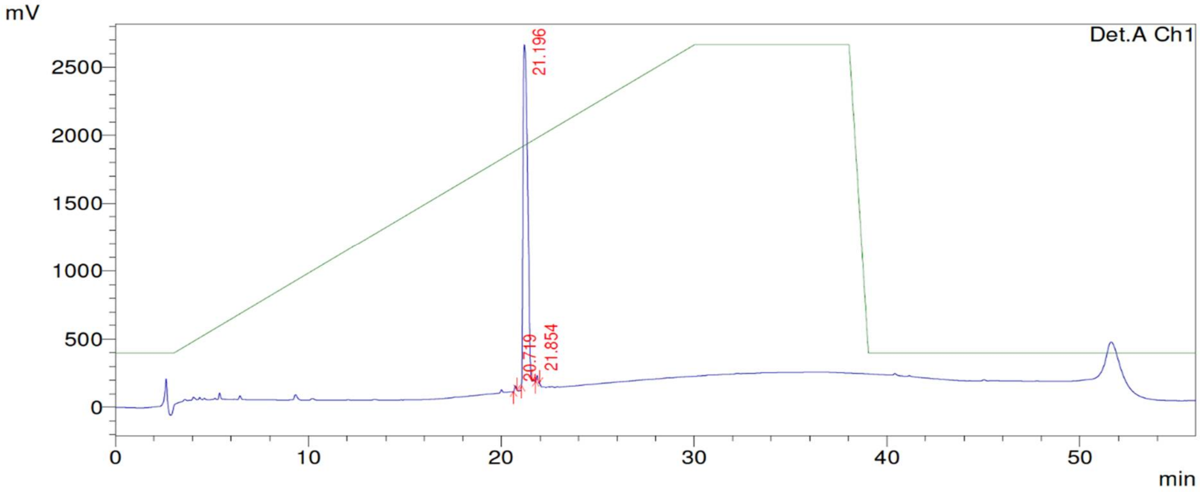

Detector A Ch1 210nm

Peak Purity Index	Ret. Time	Area	Height	Area %	Height %
	20.719	198306	38021	0.477	1.472
	21.196	41137147	2498505	98.977	96.734
	21.854	226985	46343	0.546	1.794
		41562438	2582869	100.000	100.000

Figure S14. Analytical purity of *mut4*. HPLC C12 column (Phenomenex® C12, Jupiter 4 µ Proteo, 90 Å, 250 × 4.6 mm) using a mixture of aqueous 0.1% (*v*/*v*) TFA (A) and 0.1% (*v*/*v*) TFA in acetonitrile (**B**) as the mobile phase (flow rate of 1 mL/min) and employing UV detection at 210 nm.
